# Genome-wide association study uncovers genomic regions associated with grain iron, zinc and protein content in pearl millet

**DOI:** 10.1038/s41598-020-76230-y

**Published:** 2020-11-10

**Authors:** Mahesh Pujar, S. Gangaprasad, Mahalingam Govindaraj, Sunil S. Gangurde, A. Kanatti, Himabindu Kudapa

**Affiliations:** 1 International Crops Research Institute for the Semi-Arid Tropics (ICRISAT), Patancheru, Telangana 502 324 India; 2 University of Agricultural Sciences, Shivamogga, Karnataka 577 225 India

**Keywords:** Plant breeding, Genetics, Plant sciences

## Abstract

Pearl millet hybrids biofortified with iron (Fe) and zinc (Zn) promise to be part of a long-term strategy to combat micronutrient malnutrition in the arid and semi-arid tropical (SAT) regions of the world. Biofortification through molecular breeding is the way forward to achieving a rapid trait-based breeding strategy. This genome-wide association study (GWAS) was conducted to identify significant marker-trait associations (MTAs) for Fe, Zn, and protein content (PC) for enhanced biofortification breeding. A diverse panel of 281 advanced inbred lines was evaluated for Fe, Zn, and PC over two seasons. Phenotypic evaluation revealed high variability (Fe: 32–120 mg kg^−1^, Zn: 19–87 mg kg^−1^, PC: 8–16%), heritability (h_bs_^2^ ≥ 90%) and significantly positive correlation among Fe, Zn and PC (*P* = 0.01), implying concurrent improvement. Based on the Diversity Arrays Technology (DArT) seq assay, 58,719 highly informative SNPs were filtered for association mapping. Population structure analysis showed six major genetic groups (K = 6). A total of 78 MTAs were identified, of which 18 were associated with Fe, 43 with Zn, and 17 with PC. Four SNPs viz., Pgl04_64673688, Pgl05_135500493, Pgl05_144482656, and Pgl07_101483782 located on chromosomes Pgl04 (1), Pgl05 (2) and Pgl07 (1), respectively were co-segregated for Fe and Zn. Promising genes, ‘Late embryogenesis abundant protein’, ‘Myb domain’, ‘pentatricopeptide repeat’, and ‘iron ion binding’ coded by 8 SNPs were identified. The SNPs/genes identified in the present study presents prospects for genomics assisted biofortification breeding in pearl millet.

## Introduction

Pearl millet is a climate-resilient crop that accounts for two-thirds of the global millet production. The crop covers more than 31 million hectares worldwide and is grown in more than 30 countries in the arid and semi-arid tropical as well as subtropical regions of Asia, Africa, and Latin America. In Asia, India is the largest producer of pearl millet, where it is grown on 9 million hectares with a production of 8.3 million tons^1^. In the African region, West and Central Africa has the largest area under the crop—15 million hectares—and has an annual production of 14.1 million tons. Pearl millet is a diploid (2*n* = 14) cross-pollinating crop (> 80%) with a genome size of ~ 1.79 GB^2^. Its domestication occurred in regions with low fertility soils, heat, and drought, making it naturally adapted to face the challenges associated with climate change.

Pearl millet grains are naturally nutritious and contain high fiber (1.2 g/100 g) and low starch. They are the richest source of grain Fe and Zn compared to other cereals^3^. Iron and zinc are two important micronutrients that play a vital role in human health. Iron is required for psychomotor development, maintenance of physical activity and work capacity, and resistance to infection^4^, whereas zinc is required for the growth and maintenance of the human immune system; hence it aids in both the prevention of and recovery from various diseases^5^. Apart from Fe and Zn, pearl millet is also rich in grain protein content (8–19%) that is almost at par with that in wheat (11.6 vs 11.8 g/100 g) and considerably higher than that in rice (6.8 g/100 g), sorghum (10.4 g/100 g) and maize (4.7 g/100 g)^6^. High-quality proteins are essential for the physical and mental well-being of humans, especially children^7,8^.

Diets deficient in Fe and Zn (micronutrient malnutrition) or protein alone or in combination lead to malnutrition which is also known as ‘hidden hunger’. It has been estimated that over 2 billion people across the world suffer from micronutrient deficiencies in developing countries like Africa and India^9^. Anaemia is alarmingly high, especially among pregnant women (40%) and children (42%) below 5 years^10^. In addition to this, cereal proteins deficient in essential amino acids such as methionine, lysine, and tryptophan are a matter of concern in developing countries^11^. Kwashiorkor, oedema, and marasmus are some of the severe forms of protein deficiency^12^. To combat hidden hunger, biofortification, wherein grain micronutrients along with grain protein contents are genetically enhanced through either conventional or molecular breeding, is gaining popularity. Genomics-assisted breeding holds potential for the rapid improvement of varieties using diagnostic markers^13,14^.

The wide variability for grain Fe and Zn content in pearl millet unveils the great prospect of developing biofortified pearl millet varieties and hybrids. The International Crops Research Institute for the Semi-Arid Tropics (ICRISAT) has been working towards developing biofortified hybrids and has successfully delivered high-Fe pearl millet varieties and hybrids with high yield potential in India and Africa^15^. Biofortification using conventional breeding is time-consuming and incurs a high cost in terms of screening hybrid parental lines for micronutrients and protein in every generation. Hence, it is important to develop a cost-effective strategy to improve nutritional traits in pearl millet breeding programs. Furthermore, Fe and Zn are complex traits governed by additive genes and are affected by G × E interactions. Nutritional traits are very complex and governed by a group of genes. It is a challenge to track the genomic regions/genes that are either directly or indirectly responsible for Fe and Zn loading in the grains. Genome analysis tools provide access to thousands of genomic polymorphisms, considerably broadening the ability to monitor and effectively utilize genetic diversity^16^. Quantitative trait loci (QTL) mapping based on linkage analysis provides the high power of QTL detection of a trait of interest; it has a very low mapping resolution because of the few recombination events that it takes into consideration which would ultimately lead to long linkage blocks^17^.

Advances in high throughput genotyping technologies such as genotyping-by-sequencing (GBS)^18^, DArT^19^, and GWAS have enabled the use of these powerful approaches in dissecting quantitative traits^20^. GWAS is a robust approach that has been successfully applied in the past to identify genomic regions controlling grain/kernel Fe and Zn contents in maize^21^, rice^22^, and wheat^23^. GWAS has been successfully applied in wheat and maize to identify grain PC. The availability of the draft genome of pearl millet^2^ provides the advantage of single nucleotide polymorphism (SNP) and candidate gene discovery. Single nucleotide polymorphism markers are desirable for GWAS, genomic selection, and QTL mapping^24^. GWAS exploits millions of SNPs generated across the whole genome through GBS, whole-genome re-sequencing (WGRS), DArT, and DArT seq using a diverse group of germplasm lines. GWAS is very effective in pearl millet due to faster LD-decay^2^. The discovery of SNP markers and their validation will help in developing diagnostic markers that can be deployed to develop biofortified pearl millet varieties/hybrids with elevated Fe and Zn content. This study aims to evaluate genetic variability for grain Fe, and Zn and PC among GWAS panel to discover the genomic regions associated with Fe, Zn, and PC in order to develop diagnostic markers for use in the pearl millet biofortification breeding program.

## Results

### Variability for Fe, Zn, and PC

**Figure 1:**
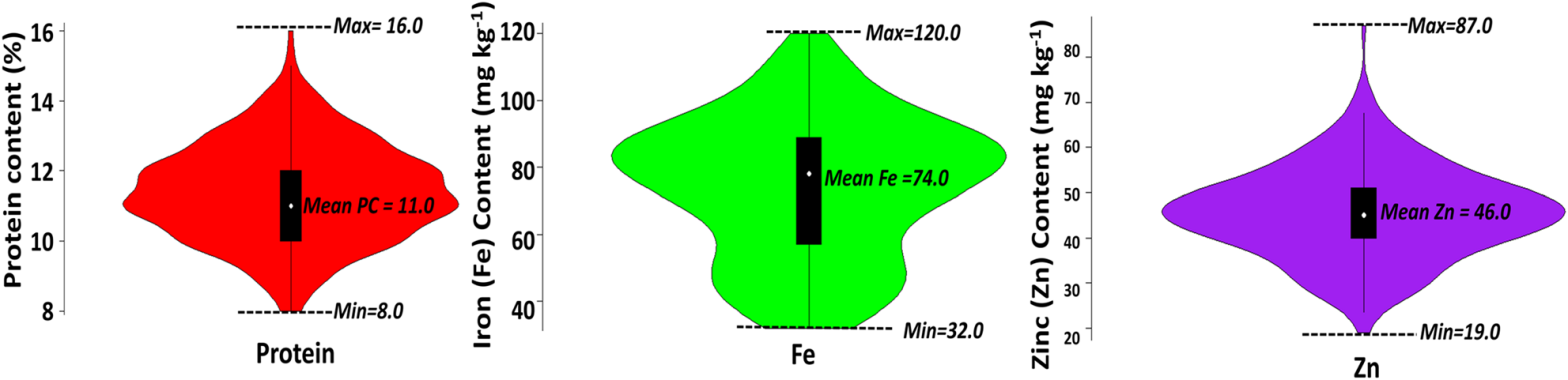
Mean, maximum and minimum for iron (Fe), zinc (Zn) and protein content (PC) among 281 inbred lines.

The analysis of variance recorded significant (*P* < 0.01) mean squares for Fe, Zn, and PC among the inbred lines. Descriptive statistics revealed the presence of significant variability (Fig. 1) with high heritability (> 90% h_bs_^2^) for three traits studied among 281 GWAS panel of pearl millet (Table 1). The Fe content in grains among inbred lines varied from 32 to 120 mg kg^−1^ with an average of 74 mg kg^−1^ (SEm = 2.72). The Zn content in grains varied from 19 to 87 mg kg^−1^ with an average of 46 mg kg^−1^ (SEm = 1.39), whereas the PC varied from 8 to 16% with an average of 11% (SEm = 3.06). Among the 281 inbred lines evaluated, 19%, 15%, and 14% of inbred lines belonging to the seed parents whereas, 24%, 18%, and 20% of inbreds belonging to restorer parents recorded higher Fe, and Zn, and PC, respectively in comparison with the overall trial mean. Furthermore, significant (*P* < 0.01) G × E interaction was recorded for all three traits. Pearson’s correlation coefficient revealed high significant (r = 0.77, *P* < 0.01) positive association between Fe and Zn, whereas PC recorded significant but moderate positive association with Fe (r = 0.38, *P* < 0.01) and Zn (r = 0.44, *P* < 0.01) (Supplementary Fig. S1).

**Table 1:** Estimates of mean, variance, range and heritability for pooled analysis of phenotypic evaluation of 281 inbred lines across 2017 rainy and 2018 summer, ICRISAT, Patancheru. CV, coefficient of variation; SEm, Standard error of mean; * and **, F-values significant at 0.05, 0.01 probability level.

Trait	Fe (mg kg^−1^)	Zn (mg kg^−1^)	PC (%)
Mean	74	46	11
Range	32–120	19–87	8–16
Heritability (h_bs_^2^) (%)	93	90	96
CV %	8.24	9.45	5.65
SE(m)	2.72	1.39	3.06
Genotype variance (${\sigma }^{2}G$)	2075.60**	545.33**	9.39**
G × E variance (${\sigma }^{2}GxE)$	166**	55.46**	3.66**
GV > GE	83%	85%	2%

### Genome-wide marker profiling

**Figure 2:**
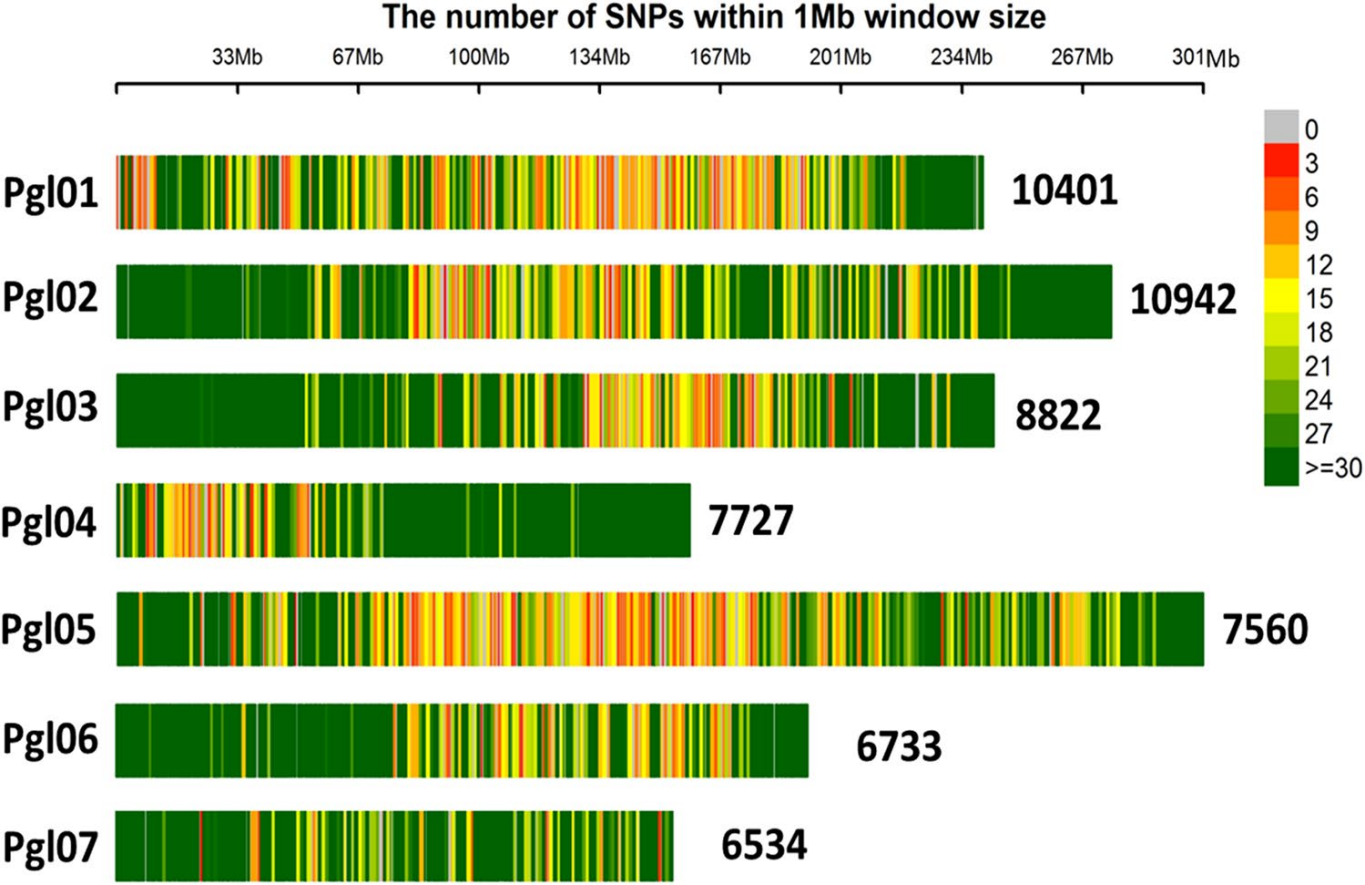
Chromosome wise distribution of 58,719 DArT-seq markers generated from genotyping by sequencing (GBS) of 281 pearl millet inbreds.

A total of 87,748 DArT seq markers were generated from the 281 GWAS panel representing restorer parents (R-lines), seed parents (B-lines), germplasm progenies and population progenies. The DArT seq markers were subjected to filtering and data quality check. All the SNP loci with > 30% missing data and rare SNPs with < 10% minor allele frequencies (MAF) were filtered and a total of 58,719 high-quality SNPs (derived from the DArT seq platform) were considered for further analysis (Fig. 2).

### Population structure and linkage disequilibrium

**Figure 3:**
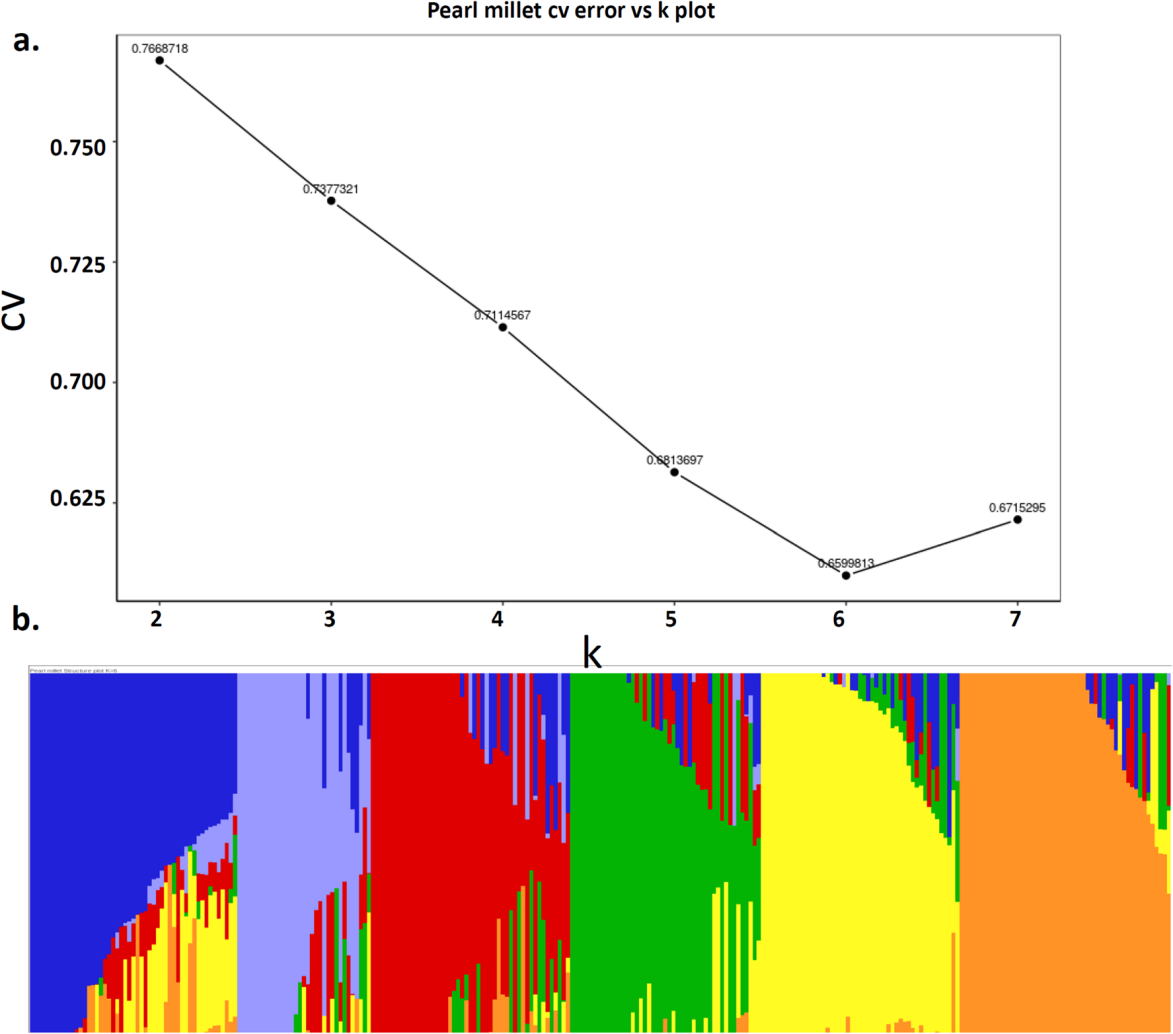
The six sub-populations of 281 pearl millet inbred lines using SNP markers (GBS-generated) in ADMIXTURE software according (Alexander et al.^73^). (**A**) Best K estimation against CV error. (**B**) Estimated population structure of 281 pearl milletinbreds as revealed by 58,719 SNP markers and K = 6. Blue, purple, red, green, yellow and brown color represents group I, II, III, IV, V and VI respectively.

Dissection of the population structure of the association panel using SNP markers revealed a total of six (K = 6) genetic groups at the corresponding least cross validation error (CV error) of 0.659 (Fig. 3A). Among the six subgroups, group VI (orange) was the largest that consisted of 53 inbreds, followed by group I (blue) with 51, group III (red) with 50, group V (yellow) with 48, group IV (green) with 47 and the group II (purple) with 32 inbred lines (Fig. 3B).

**Figure 4:**
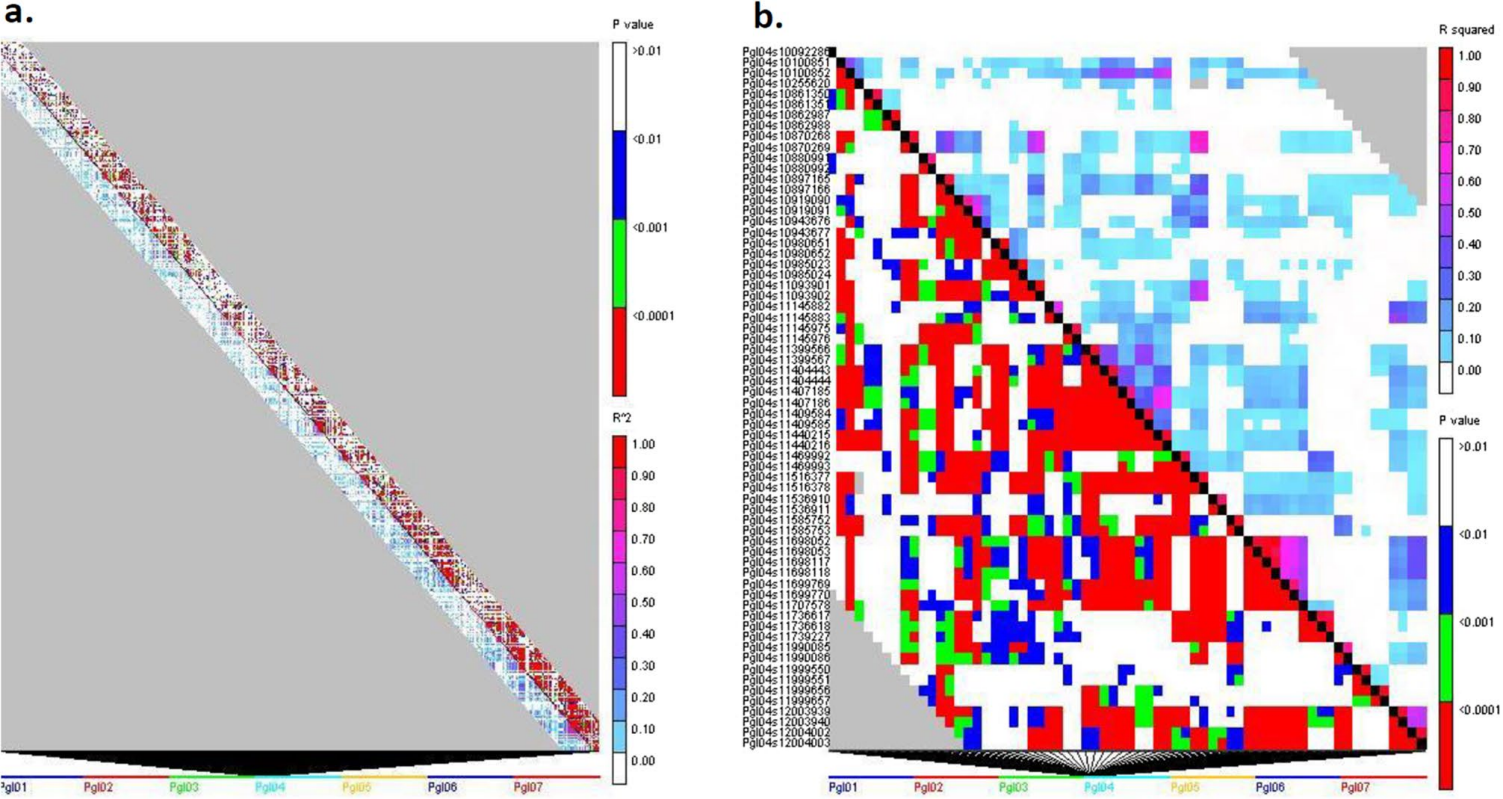
Linkage disequilibrium (LD) plot representation across each trait among seven chromosomes. (**A**) LD-plot for all the seven chromosomes. (**B**) LD-plot for only chromosome Pgl04.

**Figure 5:**
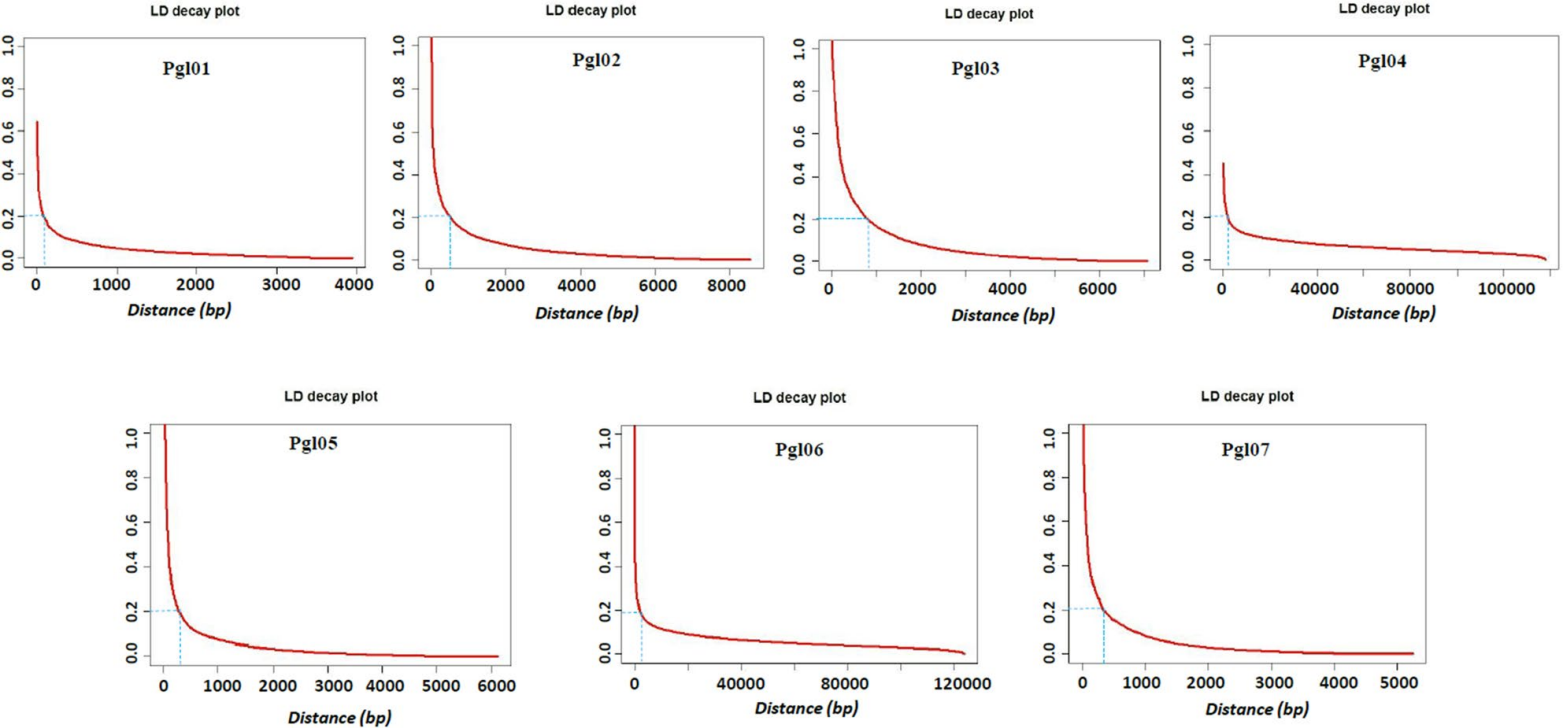
Linkage disequilibrium decay (LDD) plot across seven chromosomes of pearl millet.

The linkage disequilibrium (LD) between each pair of SNPs across each chromosome was evaluated by the squared Pearson correlation coefficient (R^2^). A set of 58,719 SNPs with identified physical positions were used for LD analysis (Fig. 4). The pairwise LD across each chromosome showed that the LD (R^2^) ranged from 0 to 1 with the average LD across the genome being 0.116. Furthermore, chromosome-wise average LD varied in the order of 0.151 > 0.138 > 0.129 > 0.118 > 0.107 > 0.087 > 0.081 for chromosomes Pgl03, Pgl07, Pgl04, Pgl06, Pgl02, Pgl01, and Pgl05, respectively. The LD for 18,80,476 pairwise combinations obtained from 58,719 marker loci across the genome showed that 57% of SNP pairs showed < 0.01 R^2^, whereas 37% of SNP pairs showed 0.01–0.05 R^2^, and only 6% of SNP pairs showed 0.06–0.1 R^2^. Linkage disequilibrium-decay (LDD) across seven chromosomes was determined using the entire set of 58,719 DArT seq markers. The LDD was plotted as LD (R^2^) between the adjacent pair of markers on the Y-axis against the distance in base pairs (bp) on the X-axis (Fig. 5). The R^2^ threshold level was set to 0.2 and observed rapid LDD across the pearl millet genome with an average LDD of 2.9 kb (2900 bp). Among the seven chromosomes, the shortest LDD was observed in chromosome 1 with 0.2 kb (200 bp, R^2^ = 0.2) and the longest LDD was observed in chromosome 6 with 9 kb (9000 bp, R^2^ = 0.2).

### Genome-wide association study

**Figure 6:**
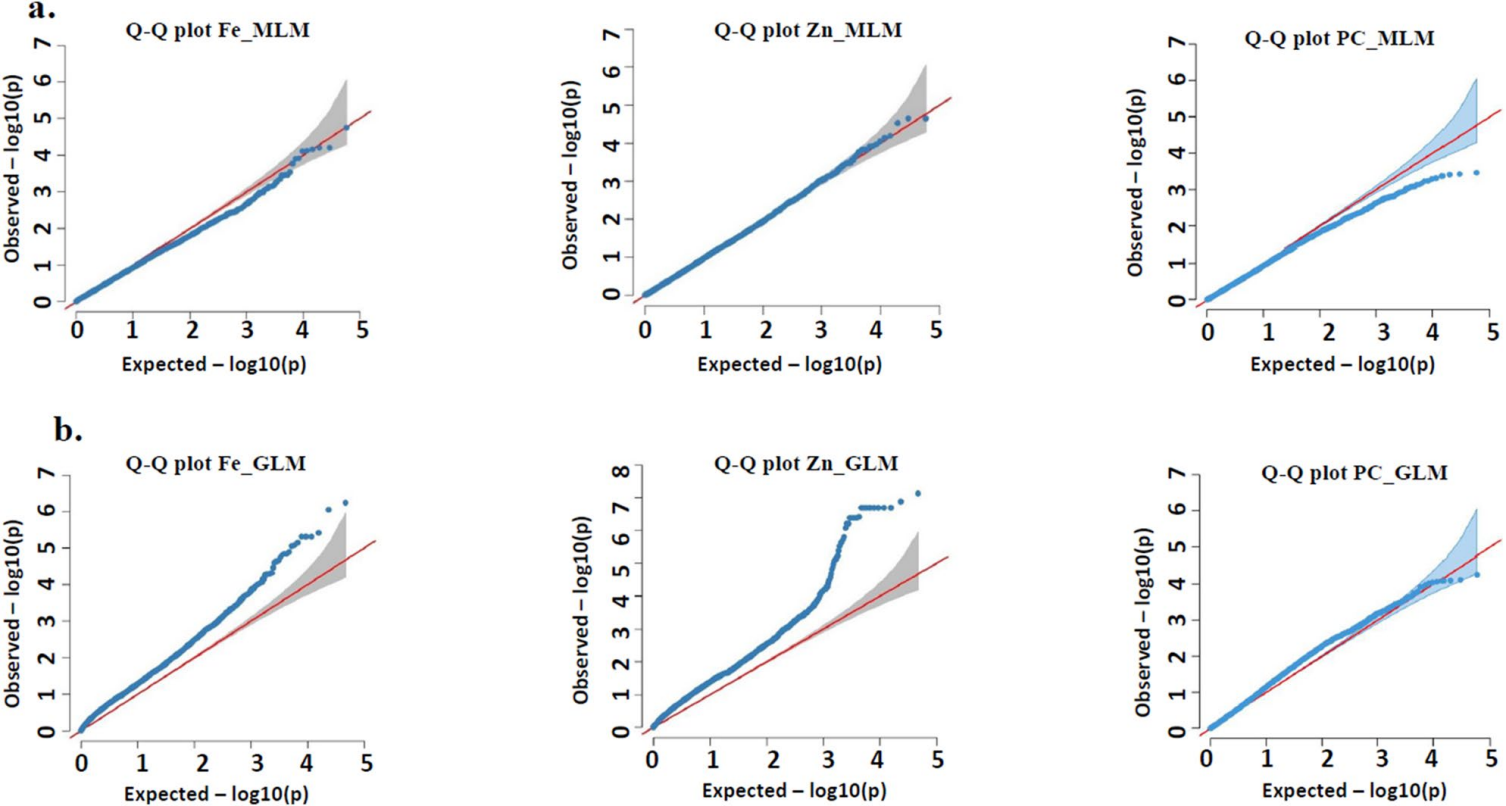
Quantile–Quantile (Q–Q) plots showing inflation of estimated − log10 (P) values versus observed for iron (Fe), zinc (Zn) and protein content (PC). (**A**) Q-Q plot for mixed linear model (MLM) and (**B**) Q–Q plot for general linear model (GLM).

A genome-wide association mapping was performed using 58,719 high-quality SNPs with less than 30% missing data having a call rate of more than 0.7. These SNPs covered around 301 Mb of pearl millet genome and were distributed across the seven chromosomes of pearl millet with a minimum of 6534 SNPs on chromosome 7 to a maximum of 10,942 SNPs on chromosome 2. SNP genotyping data of 58, 719 SNPs along with information on population structure and kinship matrix were used for genome-wide association analysis against Fe, Zn, and PC in grains for the pooled data across the 2017 rainy season and 2018 summer season. Among two models used for GWAS, the general linear model (GLM) considering only population structure (Q) showed high genomic inflation (Fig. 6), whereas the mixed linear model (MLM) which considers both population structure and family relatedness (K) showed low genomic inflation and thus helped overcome the number of false-positive associations for Fe, Zn, and PC. Therefore, significant marker-trait associations (MTAs) finalized based only on MLM are presented here. The threshold level of ‘*P*’ value was set to 3.0, above which the SNPs are said to be significantly associated. A total of 78 MTAs were identified based on their **‘P’** values. Of the 78 MTAs identified across the three traits, 16 MTAs were identified on chromosome 5 followed by 14 MTAs each on chromosome 4 and chromosome 7; 13 MTAs on chromosome 1; 10 MTAs on chromosome 2; and 3 MTAs on chromosome 3 (Supplementary Table S4 for trait-wise and chromosome-wise MTAs).

### Genomic regions identified for grain Fe and Zn content

**Figure 7:**
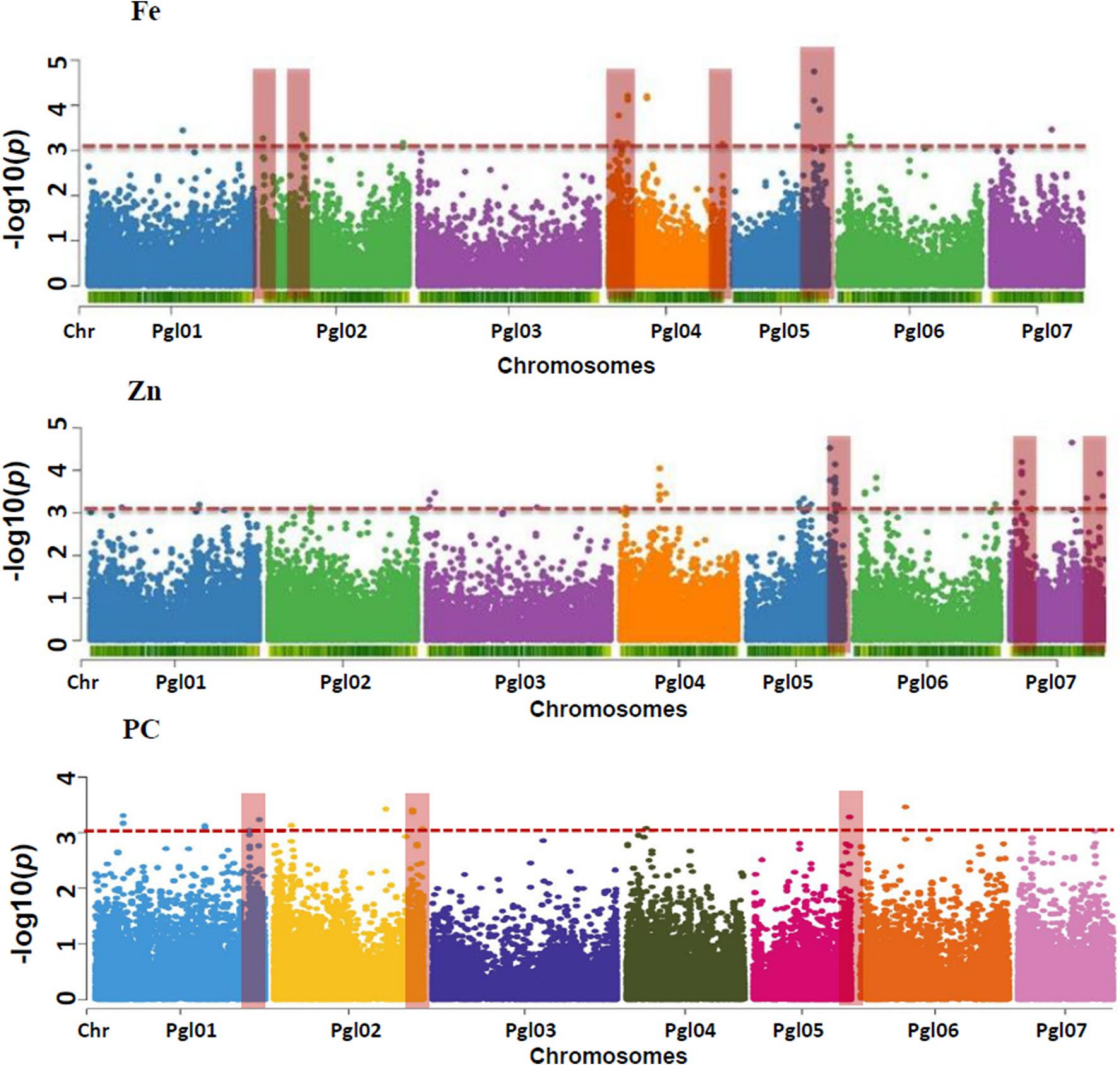
Manhattan plot from the Q + K (MLM) model for Fe, Zn, and PC plotted against individual SNPs across all chromosomes on the X-axis and − log10 P value of each SNP on the Y-axis. The different colors indicate the 7 chromosomes of pearl millet (Pgl01, Pgl02, Pgl03, Pgl04, Pgl05, Pgl06, and Pgl07). The pink dotted horizontal line shows the multiple testing threshold − log10 P value of 3 for the present GWAS panel.

A total of 61 highly significant MTAs for grain micronutrients were identified. Of the 61 MTAs, 18 were identified for Fe (Table 2; Fig. 7) with ‘*P’* values ranging from 1.79 × 10^–5^ to 9.83 × 10^–4^ which explained 5.07 to 8.23% of phenotypic variation (PVE). The 18 markers that were identified for Fe were distributed across chromosome Pgl01 (1), Pgl02 (4), Pgl04 (7), Pgl05 (3), Pgl06 (2), and Pgl07 (1). No SNPs were found associated with chromosome Pgl03. Pgl05_135500493 was identified with the highest phenotypic variation of 8.23% for Fe with a ‘*P*’ value of 1.79 × 10^–5^.

**Table 2:** Marker trait associations (MTAs) or SNPs identified for the iron (Fe), zinc (Zn) and protein content (PC) using mixed linear model (MLM) with annotations of corresponding gene.

Trait	Marker ID	Chromosome	Locus position	−Log_10_ P	*P*	R^2^/PVE	Gene annotation
Fe	Pgl01_157344213	Pgl01	157,344,213	3.12	7.58E−04	5.27	Like-Sm ribonucleoprotein (LSM)
Fe	Pgl02_8191	Pgl02	8191	3.26	5.44E–04	5.59	bZIP-1
Fe	Pgl02_64976379	Pgl02	64,976,379	3.27	5.39E–04	5.52	DNA-binding domain, Ankyrin repeat-containing domain
Fe	Pgl02_69249845	Pgl02	69,249,845	3.3	4.97E–04	5.59	Late embryogenesis abundant protein, LEA-25
Fe	Pgl02_233052877	Pgl02	233,052,877	3.05	8.85E–04	5.15	Leucine-rich repeat
Fe	Pgl04_190105720	Pgl04	190,105,720	3.14	7.25E–04	5.37	Zinc finger
Fe	Pgl04_15506741	Pgl04	15,506,741	3.01	9.83E–04	5.07	–
Fe	Pgl04_17259669	Pgl04	17,259,669	3.54	2.86E–04	6	–
Fe	Pgl04_23381732	Pgl04	23,381,732	3.14	7.27E–04	5.37	Ubiquitin-conjugating enzyme
Fe	Pgl04_32057582	Pgl04	32,057,582	3.15	7.16E–04	5.38	–
Fe	Pgl04_32617883	Pgl04	32,617,883	3.18	6.60E–04	5.37	Domain of unknown function
Fe	Pgl04_64673688	Pgl04	64,673,688	3.6	2.53E–04	6.1	–
Fe	Pgl05_107148808	Pgl05	107,148,808	3.53	2.93E–04	6.06	Cytochrome P450
Fe	Pgl05_135500493	Pgl05	135,500,493	4.75	1.79E–05	8.23	–
Fe	Pgl05_144482656	Pgl05	144,482,656	4.04	9.03E–05	6.88	–
Fe	Pgl06_21219367	Pgl06	21,219,367	3.31	4.87E–04	5.68	Oligopeptide transporter
Fe	Pgl06_145237122	Pgl06	145,237,122	3.04	9.16E–04	5.19	–
Fe	Pgl07_101483782	Pgl07	101,483,782	3.49	3.22E–04	5.91	Pentatricopeptide repeat
Zn	Pgl01_568786	Pgl01	568,786	3	9.93E–04	5.09	Heat shock protein Hsp70
Zn	Pgl01_51414126	Pgl01	51,414,126	3.38	4.14E–04	5.72	Protein kinase, catalytic domain, Leucine-rich repeat
Zn	Pgl01_172878523	Pgl01	172,878,523	3.02	9.52E–04	5.1	–
Zn	Pgl01_177992632	Pgl01	177,992,632	3.2	6.37E–04	5.43	Protein kinase, catalytic domain
Zn	Pgl01_218681895	Pgl01	218,681,895	3.44	3.65E–04	5.82	Peptidase S16
Zn	Pgl02_69256531	Pgl02	69,256,531	3.51	3.08E–04	5.95	Myb transcription factor
Zn	Pgl03_180499360	Pgl03	180,499,360	3.06	8.80E–04	5.15	–
Zn	Pgl03_4732348	Pgl03	4,732,348	3.31	4.95E–04	5.62	–
Zn	Pgl03_13329915	Pgl03	13,329,915	3.61	2.44E–04	6.12	–
Zn	Pgl04_1518626	Pgl04	1,518,626	3.2	6.38E–04	5.4	Disease resistance protein
Zn	Pgl04_9044259	Pgl04	9,044,259	3.01	9.71E–04	5.11	–
Zn	Pgl04_9059217	Pgl04	9,059,217	3.12	7.61E–04	5.3	–
Zn	Pgl04_64429980	Pgl04	64,429,980	3.04	9.02E–04	5.14	BTB/POZ-like
Zn	Pgl04_64673688	Pgl04	64,673,688	3.37	4.23E–04	5.71	–
Zn	Pgl04_74518920	Pgl04	74,518,920	3.45	3.52E–04	5.88	MATH
Zn	Pgl05_85608777	Pgl05	85,608,777	3.3	5.04E–04	5.57	Glycosyl transferase
Zn	Pgl05_91509511	Pgl05	91,509,511	3.06	8.71E–04	5.19	Oligopeptide transporter|
Zn	Pgl05_92617645	Pgl05	92,617,645	3.34	4.62E–04	5.68	–
Zn	Pgl05_92926570	Pgl05	92,926,570	3.57	2.71E–04	6.04	Protein of unknown function DUF2045
Zn	Pgl05_98096070	Pgl05	98,096,070	3.13	7.40E–04	5.32	Domain of unknown function DUF828
Zn	Pgl05_104608199	Pgl05	104,608,199	3.2	6.35E–04	5.43	Domain of unknown function DUF1618
Zn	Pgl05_135500493	Pgl05	135,500,493	3.76	1.72E–04	6.43	Glycosyl transferase, family 1
Zn	Pgl05_143124835	Pgl05	143,124,835	3.76	1.75E–04	6.41	C-5 cytosine methyltransferase
Zn	Pgl05_143702980	Pgl05	143,702,980	3.1	7.91E–04	5.23	Mini-chromosome maintenance, DNA-dependent ATPase
Zn	Pgl05_143706557	Pgl05	143,706,557	3.53	2.93E–04	5.98	Mini-chromosome maintenance, DNA-dependent ATPase
Zn	Pgl05_144482656	Pgl05	144,482,656	3.05	8.82E–04	5.18	–
Zn	Pgl05_148964458	Pgl05	148,964,458	3.21	6.15E–04	5.42	Ribosomal protein L10/acidic P0
Zn	Pgl06_223926259	Pgl06	223,926,259	3.01	9.83E–04	5.1	–
Zn	Pgl06_231796045	Pgl06	231,796,045	3.2	6.28E–04	5.44	SANT/Myb domain
Zn	Pgl06_18558795	Pgl06	18,558,795	3.48	3.29E–04	5.93	RNA methyltransferase, RsmD
Zn	Pgl06_36628895	Pgl06	36,628,895	3.82	1.50E–04	6.53	–
Zn	Pgl06_54978917	Pgl06	54,978,917	3	9.98E–04	5.09	Resolvase, holliday junction-type, YqgF-like
Zn	Pgl07_9399240	Pgl07	9,399,240	3.24	5.69E–04	5.52	GRAM
Zn	Pgl07_19060446	Pgl07	19,060,446	3.91	1.23E–04	6.69	Blue (type 1) copper domain
Zn	Pgl07_19133990	Pgl07	19,133,990	3.09	8.12E–04	5.21	Peptidase S8/S53, subtilisin/kexin/sedolisin
Zn	Pgl07_20613468	Pgl07	20,613,468	3.47	3.40E–04	5.91	–
Zn	Pgl07_35376984	Pgl07	35,376,984	3.1	8.03E–04	5.26	Male sterility, NAD-binding
Zn	Pgl07_101483782	Pgl07	101,483,782	4.65	2.24E–05	8	Pentatricopeptide repeat
Zn	Pgl07_101483780	Pgl07	101,483,780	4.55	2.85E–05	7.76	Pentatricopeptide repeat
Zn	Pgl07_101517680	Pgl07	101,517,680	3.2	6.33E–04	5.4	Chalcone/stilbene synthase, C-terminal
Zn	Pgl07_125865145	Pgl07	125,865,145	3.34	4.57E–04	5.68	Disease resistance protein
Zn	Pgl07_147179490	Pgl07	147,179,490	4.43	3.70E–05	7.56	–
Zn	Pgl07_151365061	Pgl07	151,365,061	3.39	4.10E–04	5.77	–
PC	Pgl01_44640725	Pgl01	44,640,725	3.31	4.88E–04	5.6	–
PC	Pgl01_44640726	Pgl01	44,640,726	3.17	6.77E–04	5.35	von Willebrand factor, type A
PC	Pgl01_177992633	Pgl01	177,992,633	3.13	7.39E–04	5.29	Homeodomain
PC	Pgl01_177992634	Pgl01	177,992,634	3.09	8.16E–04	5.21	Protein kinase, catalytic domain
PC	Pgl01_250761833	Pgl01	250,761,833	3.04	9.04E–04	5.13	Protein kinase, catalytic domain
PC	Pgl01_266542617	Pgl01	266,542,617	3.24	5.80E–04	5.47	–
PC	Pgl01_266542615	Pgl01	266,542,615	3.23	5.87E–04	5.46	von Willebrand factor, type A
PC	Pgl02_28323518	Pgl02	28,323,518	3.13	7.35E–04	5.29	–
PC	Pgl02_182371002	Pgl02	182,371,002	3.42	3.77E–04	5.79	–
PC	Pgl02_225493497	Pgl02	225,493,497	3.37	4.24E–04	5.7	–
PC	Pgl02_225493495	Pgl02	225,493,495	3.41	3.92E–04	5.76	–
PC	Pgl02_241839676	Pgl02	241,839,676	3.07	8.43E–04	5.19	Cytochrome P450
PC	Pgl04_32176024	Pgl04	32,176,024	3.08	8.26E–04	5.2	Protein kinase, catalytic domain
PC	Pgl05_156574366	Pgl05	156,574,366	3.28	5.21E–04	5.55	–
PC	Pgl06_71295563	Pgl06	71,295,563	3.46	3.46E–04	5.86	–
PC	Pgl07_124769335	Pgl07	124,769,335	3.03	9.39E–04	5.11	–
PC	Pgl07_124769336	Pgl07	124,769,336	3.03	9.39E–04	5.11	Domain of unknown function DUF547

However, a total of 43 significantly associated markers were identified for Zn with ‘*P’* values ranging from 2.24 × 10^–5^ to 9.78 × 10^–4^. Furthermore, the phenotypic variation explained by these SNPs ranged from 5.09 to 8.00% for Zn. These 43 markers identified were distributed across chromosomes Pgl01 (5), Pgl02 (1), Pg103 (3), Pgl04 (6), Pgl05 (12), Pg106 (5) and Pgl07 (11), respectively. Pgl07_101483782 for Zn was identified with the highest phenotypic variation of 8.00% with a ‘*P’* value of 2.24 × 10^–5^. A total of four SNPs (Pgl04_64673688, Pgl05_135500493, Pgl05_144482656 and Pgl07_101483782) located on three different chromosomes (4, 5 and 7) were found common among grain Fe and Zn contents (Supplementary Table S2).

### Grain protein content (PC)

A total 17 MTAs were identified for PC with ‘*P’* values ranging from 3.46 × 10^–4^ to 9.39 × 10^–4^, which explained 5.11 to 5.68% of the phenotypic variation. The 17 markers that were identified for PC were distributed across chromosomes Pgl01 (7), Pgl02 (5), Pgl04 (1), Pgl05 (1), Pgl06 (1), and Pgl07 (2). No SNPs were found associated with chromosome Pgl03. Pgl06_71295563 was identified with the phenotypic variation of ~ 6% for PC with a ‘*P*’ value of 3.46 × 10^–4^.

### Candidate genes associated with grain Fe, Zn, and PC

Pearl millet genome sequencing reported a total of 69,398 genes and unraveled the involvement of several genes in the control of both agronomically and nutritionally important traits. The physical positions of each SNP marker from the present study were compared against the pearl millet genome sequence to determine the function of the gene underlying the respective SNP. A total of 18 SNPs associated with Fe were found linked (Table 2 and Supplementary Table S3) to different genes viz., Like-Sm ribonucleoprotein (LSM) domain, late embryogenesis abundant protein, zinc finger, ankyrin repeat, leucine-rich repeat, pentatricopeptide repeat, oligopeptide transferase, and basic leucine zipper which were found to play a significant role in plant metabolism, including iron homeostasis. Similarly, the SNPs associated with the genes viz., protein kinase, Myb transcription factor, glycosyl transferase, chalcone/stilbene synthase, heat shock protein (HSP70), peptidase, copper domain, male sterility, etc., were found to be unique to Zn while protein binding, lipid binding, protein kinase activity, and iron ion binding genes were found associated with SNPs identified for PC.

## Discussion

Developing biofortified hybrids in pearl millet requires high Fe and Zn content in both the parents since it’s governed by additive gene^25^. It is highly feasible to develop biofortified inbred lines through inbreeding which accumulates more of additive variances in subsequent generations. The strong epigenetic influence on these traits expression and sample contamination during handling of breeding materials is a challenge for biofortification in pearl millet^26,27^. The process of identifying molecular markers, preferably SNPs tightly linked to genomic regions of Fe, Zn and PC, will enhance the efficiency of biofortification using genomics assisted breeding. Recently, several genomic regions controlling the inheritance of Fe and Zn have been identified through QTL mapping^28^ using DArT and SSR markers and also through LD-based association mapping^29^ by SSR markers in pearl millet. Though SSRs are preferred markers, their resolution is relatively low^17^. None of the previous studies have reached the gene level; therefore, the present study aimed to dissect the genetic nature of Fe, Zn and PC in pearl millet using GWAS by exploiting the DArT seq markers to discover the genomic regions and candidate genes influencing Fe, Zn and PC.

Grain Fe and Zn content are strongly influenced by the available Fe and Zn content in the soil. The available soil Fe and Zn content in our experimental field was above the critical levels (2.6 to 4.5 mg kg^–1^ Fe and 0.6 to 1.0 mg kg^–1^ Zn) required for normal growth and development^30,31^. Three to fourfold significant variations for Fe (32–120 mg kg^−1^), Zn (19–87 mg kg^−1^) and twofold variation for PC (8–16%) in 281 elite inbred lines prospects the breeding feasibility (Supplementary Table S5). Similar variability for Fe/Zn has been reported among germplasm^32^, breeding lines^15^, and commercial cultivars^33^. High genetic variance for Fe/Zn indicates the least influence of G × E. Population structure along with shared co-ancestry coefficients between individuals of subdivisions of a population were estimated using ADMIXTURE 1.23^73^. A total of six genetic groups were formed among 281 inbred lines with some admixtures indicating common allelic combinations in the genomic background of few genotypes. The availability of six subgroups and wide phenotypic variation observed for Fe, Zn, and PC indicated that the present GWAS panel is best suited for genome-wide association study to dissect the genetic basis of high Fe, Zn accumulation, and PC in pearl millet.

LD is the non-random association of alleles at two or more loci and acts as a critical genetic force in determining population structure^34,35^. The LD of a population is the result of evolutionary changes in a population that would help in mapping quantitative traits such as Fe, Zn and PC more precisely while it also gives insights into the joint evolution of the linked sets of genes. The pattern of LD across the genome ultimately decides the success of association studies^36,37^. In the present study, the average pairwise LD (R^2^) across the genome decreased rapidly against the increasing distance (bp). Rapid LDD has been reported in earlier studies in pearl millet^2,38^. Chromosomes Pgl01, Pgl02, Pgl03, Pgl05, and Pgl07 showed relatively more rapid LDD (~ 0.64 kb) compared to Pgl04 and Pgl06, suggesting that a larger number of markers are required for chromosomes Pgl01, Pgl02, Pgl03, Pgl05, and Pgl07 for GWAS. The gene-rich genomic region tends to have a higher rate of recombination. Thus the LDD would be higher in such genomic regions, requiring a higher marker density for LD analysis in such regions. Of 18,80,476 pairwise LD analysis, 57% of the SNP pairs showed an LD of less than 0.01 (R^2^ = 1%), indicating that the LD in the current GWAS panel is relatively low. This could probably be because pearl millet is a highly cross-pollinated (> 80%) species, wherein some portion of the genome is bound to have heterozygosity (not every locus is heterozygous) as genetic load by the inbreeding process^39^. The low LD is also due to frequent recombination and higher inbreeding depression by virtue of being a cross-pollinated crop. The low value of LD in turn gives the high resolution of mapping but requires a large number of markers^40^.

While performing GWAS, care should be taken to avoid false associations arising from false positives (Type I error). In the present study, two extensively used statistical models, GLM^41^ and MLM^42,43^, were used for the MTA. The MLM model is more efficient and superior in reducing false positive associations by correcting for both population structure (Q) and kinship matrix (K) which can be further visualized through Quantile–Quantile (Q–Q) plots to show low genomic inflation for MLM compared to GLM (Fig. 6). However, sometimes MLM tends to overcompensate for both population structure and kinship, which could lead to false negatives, type II errors^44,45^. This means that the identification of some MTAs depends on the model used^46^. Therefore, the present study used both GLM and MLM and found that > 70% of SNPs from MLM were common in GLM, with some additional markers that were absent in GLM. Therefore, the results obtained from the MLM model are presented. None of the MTAs met the Bonferroni criteria because of the utilization of 0.058 Million markers generated through the GBS method. The Bonferroni correction would be too stringent to use as not all the markers are independent^47^ and may lead to false negatives^48,49^.

Among the significantly associated SNPs for Fe, marker Pgl05_135500493 on chromosome Pgl05 explained the highest phenotypic variation (8.23%). For Zn, markers Pgl07_101483782, Pgl07_101483780, and Pgl07_147179490 exhibited more than 7.5% of phenotypic variation. However, the SNPs identified for PC explained the relatively lower phenotypic variation, wherein the highest phenotypic variation was explained by the SNP Pgl07_71295563 on chromosome Pgl07 (~ 6%). Interestingly, there were four SNPs discovered to be common for both Fe and Zn content on chromosomes Pgl04, Pgl05, and Pgl07 that cumulatively explain about 27.12% and 25.32% of phenotypic variation for Fe and Zn, respectively. The co-localization of both Fe and Zn and highly significant positive correlation between them further suggested some common genes and pathways involved in Fe and Zn homeostasis in plants i.e., from root absorption to till deposition in grains. A common set of markers for Fe and Zn has been reported in pearl millet^29^ on LG 3, LG 5, and LG 7. QTLs responsible for Fe and Zn have been co-mapped on LG 1 and LG 7^28^; these probably indicate that chromosome Pgl05 and Pgl07 are likely to control Fe and Zn transport and accumulation in pearl millet. Though no common MTAs were identified for PC with Fe and Zn, the positive significant correlation of PC with both Fe and Zn suggested that the selection for high Fe/Zn expected to increase PC as an associated trait.

To know the conformity of the identified MTAs in this study, they were compared to previous genetic mapping studies for Fe and Zn in pearl millet. SNPs were identified for Fe and Zn in this study were concomitant to reported studies in pearl millet (Table 3). For instance, Anuradha et al.^29^ reported that Fe was highly influenced by the genes on chromosomes Pgl05 and Pgl07, whereas Kumar et al.^28^ identified genomic regions for Zn on chromosomes Pgl01 and Pgl04 in pearl millet. Zn content was also influenced by the SNPs on chromosome Pgl03, Pgl04, Pgl05, Pgl06 and Pgl07. Similar results were reported earlier by Anuradha et al.^29^ while Kumar et al.^28^ reported genomic regions on LG 1, 4, 5, and 7. This evidence suggests that the SNPs identified on chromosomes were consistent with the previously reported markers which might have a significant role to play in the expression of Fe and Zn content. This calls for fine mapping of these genomic regions that would ultimately provide candidate SNPs for use in marker-assisted breeding to improve grain Fe and Zn. Apart from pearl millet, genomic regions were also discovered for grain Fe and Zn content in other millets and cereals such as rice^22^, foxtail millet^50^, maize^21^, wheat^23^, through genome-wide association mapping. Genetic mapping studies have discovered genomic regions for grain Fe and Zn content in sorghum^51^, maize^52^, and wheat^53^. Hence different genomic regions in this study can be introgressed for trait improvement in pearl millet based on the targeted environment, depending on common MTAs. This is the first report on the discovery of genomic regions using GWAS for PC in pearl millet. The findings will generate research interest to further investigate the regulation of grain PC in pearl millet. A total of 17 MTAs were identified on six chromosomes (Pgl01, Pgl02, Pgl04, Pgl05, Pgl06 and Pgl07) of pearl millet, among which Pgl06_71295563 showed the highest phenotypic variation of 5.86% with a *‘P’* value of 3.46 × 10^4^. Similar genomic regions have been reported for PC in previous studies in maize^54^, rice^55,56^, and wheat^57^.

**Table 3:** QTLs reported from earlier studies for iron (Fe), zinc (Zn) in pearl millet and co-localized associated marker trait associations (MTAs) identified the same genomic region in present study.

SN	Genetic mapping	Trait	Linkage group (LG)/chromosome	MTAs on respective chromosomes from current study	Author
1	Association mapping	Fe	**LG3, LG5, LG7**	Pgl05_144482656, Pgl05_144482654, Pgl05_148774199, Pgl05_148774200, Pgl07_101483782	Anuradha et al.^29^
2	QTL-map	Fe	**LG 1,3,7**	Pgl01_157344213, Pgl01_157344211	Kuamar et al.^28^
3	Association mapping	Zn	**LG3, LG4, LG5, LG6,LG7**	Pgl03_180499360, Pgl03_251188374, Pgl03_13329915, Pgl04_1518626, Pgl04_64429980, Pgl04_64673688, Pgl05_85608777, Pgl05_92617645, Pgl05_92926570, Pgl05_135474055, Pgl05_135500493, Pgl05_143124835,, Pgl05_143702980, Pgl05_143706557, Pgl05_144482656, Pgl05_148964458, Pgl06_12389662, Pgl06_36628894, Pgl06_119701975, Pgl07_19133990, Pgl07_20613468, Pgl07_101483780, Pgl07_101517680, Pgl07_147179490, Pgl07_151365061	Anuradha et al.^29^
4	QTL-map	Zn	**LG 1,4,5,7**	Pgl01_51414126, Pgl01_97166555, Pgl01_172878523, Pgl01_218681896, Pgl01_218681895, Pgl01_256038591, Pgl01_260361246	Kumar et al.^28^

Gene annotation was performed by comparing the sequence reads of significantly associated SNPs at their respective physical positions against the reference genome of pearl millet. The genes identified in the present study and their functional roles in Fe and Zn metabolism in plants reported through previous studies are presented in Table 4. There were several genes identified, among which very few were involved in Fe transportation, accumulation, and homeostasis. The SNP Pgl07_147858723 corresponding to glutathione S-transferase plays a significant role in iron starvation in roots. In the roots of hexaploid wheat, a significant temporal increase in glutathione S-transferase was observed at both transcriptional and enzymatic activity levels, which established the foundation for designing breeding strategies to improve Fe nutrition in pearl millet. The SNP Pgl02_69256531 and Pgl06_231796045 were found in the region of the MYB-domain. Palmer et al*.*^58^ observed that the MYB-domain plays a significant role in plant survival under Fe deficiency conditions, and is the most highly induced transcription factor which acted early in the Fe deficiency regulatory cascade to drive gene expression of *NAS4.* Shen et al.^59^ isolated MYB gene MxMYB1 from *Malus xiaojinensis*. The expression of MxMYB1 was up-regulated by Fe starvation in the roots but not in the leaves, signifying that MxMYB1 likely to play more in iron absorption from soil to roots and not likely from root to leaves. The SNP Pgl04_190105720 corresponding to the Zinc finger plays a crucial role in preventing toxic ion damage and hence performs an important role in maintaining cellular osmotic adjustment and enzyme activities, leading to significantly improved salt stress tolerance^60^.

**Table 4:** List of trait wise marker trait associations (MTAs) annotated in the present study and their respective role in iron (Fe) metabolism reported earlier in other crops.

Trait	Marker	Position	Annotations	Function reported earlier	Crop	References
Fe	Pgl02_69249845	69,249,845	IPR005513; Late embryogenesis abundant protein, LEA-25/LEA-D113	Transport of Fe in Phloem	Castor bean (*Ricinus communis*)	Kruger et al.^80^
Fe	Pgl07_101483782	101,483,782	IPR002885; Pentatricopeptide repeat	Fe homeostasis	Higher plants (*Schizosccharomyces pombe*)	Su et al.^81^
Zn	Pgl02_69256531	69,256,531	IPR001005; SANT/Myb domain|IPR015495; Myb transcription factor|IPR017930; Myb domain, DNA-binding	Iron uptake and homeostasis	Arabidopsis (*Arabidopsis thaliana*)	Chen et al.^82^, Palmer et al.^58^
				Iron root nutrition	*Malus xiaojinensis*	Shen et al.^59^

The significant phenotypic variability observed in the association panel coupled with high marker density across all chromosomes provided a strong case for whole-genome association mapping of the three (Fe, Zn, and PC) important nutritional traits in pearl millet. This GWAS study which identified marker-trait associations for Fe, Zn, and PC using the genotyping-by-sequencing platform presents greater prospects for utilization and traits mainstreaming. Rapid LDD observed in the current GWAS panel indicates that the SNPs identified through genome-wide association mapping are more reliable and complement previously reported QTLs in pearl millet. Pgl05_135500493 and Pgl05_144482656 SNPs for Fe; Pgl07_101483782, Pgl07_101483780 and Pgl07_147179490 SNPs for Zn, and Pgl06_71295563 SNPs for PC were found promising. Significant phenotypic correlations between Fe and Zn support simultaneous selection and improvement. This linkage and the identified co-localized MTA suggest there is a common physiological pathway. These MTAs help to move towards fine mapping and discovering a set of diagnostic markers to screen segregating population (F_2_/F_3_s) in order to avoid expensive phenotyping and G × E effects in future. Eight MTAs that were identified for Fe and Zn were found to be involved in Fe mobilization. Thus, the promising MTAs identified in the present study merit further validation in different genetic backgrounds of breeding lines and populations. Eleven inbred lines had ≥ 80 mg kg^−1^ of Fe, > 60 mg kg^−1^ Zn, and > 13% of PC that meet global targets and will serve as trait sources in elite backgrounds. Such lines will be easily converted into CMS (maintainers) to make hybrids with high-Fe/Zn/PC restorers for fast-track product development. The inbred panel studied that is part of hybrid parents at ICRISAT. This will enhance the introgression of these traits to develop high-yielding hybrids through marker-assisted back-crossing (MABC) in India where hybrid cultivars are dominant, while in Sub-Saharan Africa where open-pollinated varieties (OPVs) are predominant, it will be done through marker-assisted recurrent selection (MARS) and marker-assisted population improvement (MAPI).

## Materials and methods

### Plant material

The GWAS panel comprised of 281 inbred lines developed at ICRISAT, Hyderabad, India, differing in grain Fe and Zn as well as agronomic traits such as flowering, plant height, tillering, panicle size, 1000-grain weight, and grain yield. The inbred lines included 112 restorer parents (R-lines), 110 seed parents (B-lines), 32 advanced progenies derived from breeding population/composites, and 27 direct derivatives of germplasm accessions (Supplementary Table S1).

### Field trials and agronomic practices

The trials were planted in alpha lattice experimental design with three replications in two contrasting environments, rainy season 2017 and summer season 2018 at ICRISAT, Hyderabad (17.53° N; 78.27°E). Each replication comprised of 20 incomplete blocks with 10 entries in each block, and every entry planted in two rows of 2 m length. Sowing was done by tractor-mounted 4-cone planter (7100 US model) with a spacing of 75 cm between rows during the rainy season 2017 and 60 cm in the summer season 2018. Overplanted plots were thinned 15 days after sowing to single plants spaced 15 cm apart within each row. A basal dose of 100 kg ha^−1^ of diammonium phosphate (18% N and 46% P) was applied at the time of field preparation and 100 kg ha^−1^ of urea (46% N) was applied as top dressing within 2 to 4 days of thinning. The trial was irrigated at 7–10 days intervals during the summer season 2018 and as required during the rainy season 2017 to avoid moisture stress. All the recommended agronomic practices were followed for good and healthy crop growth. Observations were recorded for five random plants per plot in each replication for Fe, Zn, and PC.

### Estimation of grain iron and zinc content

For grain sampling, open-pollinated main panicles from five representative plants per plot were harvested at physiological maturity (85–90 days after planting). These panicles were stored separately in a cloth bag and sundried for 10 to 15 days, and then hand threshed to produce clean grain samples for micronutrient analysis (Fe and Zn). Utmost care was taken to avoid contact with iron equipment while threshing and handling of threshed samples. Grain Fe and Zn content were analyzed using Inductively Coupled Plasma Optical Emission Spectrometry (ICP-OES) at Flinders University, Australia, following the method described by Wheal et al*.*^61^. Grain samples were finely ground and oven-dried at 60 °C for 48 h before analyzing them for Fe and Zn. A ground sample of 0.2 g was transferred into 25 ml polyprophelene PPT tubes with 2.0 ml of concentrated nitric acid (HNO_3_) and 0.5 ml of 30% hydrogen peroxide (H_2_0_2_). These samples were wetted and predigested overnight at room temperature. Samples were placed in the digestion block and heated at 80 °C for 1 h, followed by digestion at 120 °C for 2 h. After digestion, each sample digest was turned into 25 ml using distilled water. The digests were filtered using Whatman no.1 filter paper and the filtrate was used to estimate Fe and Zn content using ICP-OES.

### Estimation of grain protein content

Grain protein content was analyzed using Near-Infrared Spectroscopy (NIRS) at ICRISAT. The quantified grain protein^62^ content was measured in percentage. The grain samples collected were cleaned thoroughly and about two to three grams of whole grain samples were poured in a small cup. The cup was then placed in the NIRS machine and the sample was run for a minute. The readings were then noted.

### Estimation of Fe and Zn content from the soil

The soil samples collected from the top 30 cm layer in the field were analyzed for extractable Fe and Zn content by Atomic Absorption Spectroscopy (AAS)^63^. The mean soil Fe and Zn content extractable with Diethylene Triamine Pentaacetic Acid (DTPA) were 3.8 mg kg^−1^ and 2.0 mg kg^−1^ during the rainy season 2017 and 5.0 mg kg^−1^ and 1.6 mg kg^−1^ during the summer season 2018, respectively.

### DNA extraction and genotyping using DArT seq

Genomic DNA was isolated from tender leaf tissues of 30 day-old seedlings^64^. The quality and quantity of the extracted DNA were checked on 0.8% agarose gel using gel electrophoresis at 80 V using λ-DNA standards. The DNA was subsequently diluted to a volume 50 μl of concentration 50 ng/μl. The samples were then sent to the Diversity Arrays Technology (DArT) Pty Ltd, Australia^65^ for genotyping using DArT markers. The DArT seq assay, an efficient genotyping-by-sequencing platform was employed in the present study. In brief, the DNA samples were digested and ligated primarily with two different adaptors accompanying to overhang by two different restriction enzymes^66^. The Illumina flow cell attachment sequence, sequencing primer sequence, and varying length barcode regions were included while designing the *PstI*-compatible adapter. The *PstI-MseI* fragments were amplified for 30 Polymerase Chain Reaction (PCR) cycles using the following reaction conditions: 94 °C for 1 min, followed by 29 cycles of 94 °C for 20 s (s), ramp 2.4 °C/s to 58 °C, 58 °C for 30 s, ramp 2.4 °C/s to 72 °C, 72 °C for 45 s. Amplicons were held at 72 °C for 7 min and then at 10 °C. All PCR amplicons from the 96-well were multiplexed in equimolar amount and kept to c-Bot (Illumina) bridge PCR after that sequenced on Illumina Hiseq2000. Single lane sequencing was followed for all the amplicons; the single read sequencing was run for 77 cycles. All the generated sequences from each lane were subjected to proprietary DArT analytical pipelines. Poor-quality sequences were filtered out from the FASTQ files in the primary pipeline. In the barcode region, more stringent selection criteria (≥ Phred pass score of 30) were employed in comparison with the rest of the sequence. The sequence assignments are authenticated to specific samples. In marker aligning, about 2,000,000 identified sequences per barcode/sample were used. Finally, identical sequences were broken into FASTQ call files. In the secondary proprietary pipeline of DArT P/L, the FASTQ call files were used to detect presence/absence markers (PAM) through SNP calling algorithms (DArTsoftseq)^67,68^.

### SNP filtering and quality control

Whole-genome genotyping data of 87,748 DArT seq markers on 281 pearl millet inbreds was generated using DArT genotyping platform. DArT seq SNP-derived markers were further filtered to remove SNPs of low quality with > 30% missing data and rare SNPs with < 10% MAF using TASSEL v 5.3.1 (Trait Analysis by Association Evolution and Linkage).

### Phenotypic data analysis

The analyses of variance was performed over the rainy season 2017 and summer season 2018 using generalized linear model procedures following a random-effects model^69,70^ in SAS University Edition (SAS/STAT, SAS Institute Inc, NC, USA)^71^. Heritability^72^ was determined using the following formula:

$${\text{H }} = \frac{{\upsigma _{{\text{g}}}^{2} }}{{\left( {\upsigma _{{\text{g}}}^{2} + \frac{{\upsigma _{{{\text{gs}}}}^{2} }}{{\text{s}}} + \frac{{\upsigma _{{\text{e}}}^{2} }}{{{\text{rs}}}}} \right)}}$$

where ${\upsigma }_{{\text{g}}}^{2}$ is the genotypic variance, ${\upsigma }_{{{\text{gs}}}}^{2}$ is the genotype × season interaction variance, and ${\upsigma }_{\mathrm{e}}^{2}$ is the residual variance; ‘r’ is the number of replications, and ‘s’ is the number of seasons. Mean and coefficient of variation (CV) were also determined using the standard procedure implemented in the SAS University Edition. Pearson’s correlation coefficients among the traits were calculated using the PROC CORR procedure in R version 3.5.1 (R Project for Statistical Computing, (https://www.r-project.org). The standard error of the mean (SEm) was determined in a simple excel program using the following formula:

$${\text{SEm}} = \sqrt {\frac{{{\text{MSS}}}}{{\text{n}}}}$$

where ‘MSS’ is the Mean sum of square and ‘n’ is the number of samples.

### Population structure, kinship and genome-wide linkage disequilibrium

Population structure was determined using ADMIXTURE 1.23 software^73^. The number of genetic clusters (K) was predefined as 1 to 10 to explore the population structure of the tested accessions. This analysis provided maximum likelihood estimates of the proportion of each sample derived from each of the K populations. The optimum K value was selected based on the graph plotted using the respective K value from 1 to10 against cross-validation error (CV-error). The optimal number of sub-population (K) was determined with the lowest cross-validation error. Genetic relatedness or K matrix was generated from TASSEL V 5.3.1.^74^. LD was quantified as adjacent pairwise R^2^ values (the squared allele frequency correlations among alleles at two adjacent SNP markers)^75^ and was estimated for 58,719 SNPs in TASSEL V 5.3.1.

### Genome-wide association analysis

Marker trait association was performed using two different models, GLM and MLM, as given below^76^:

$$\begin{aligned} & {\text{y }} = {\text{ X}}_{{\text{a}}} + {\text{ Q}}_{{\text{b}}} + {\text{ e}}\quad \quad {\text{GLM and}} \\ & {\text{y }} = {\text{ X}}_{{\text{a}}} + {\text{ Q}}_{{\text{b}}} + {\text{ Z}}_{{\text{u}}} + {\text{ e}}\quad \quad {\text{MLM}} \\ \end{aligned}$$

where, ‘y’ is phenotype vector, ‘a’ is a marker vector with fixed effects, ‘b’ is a vector with fixed effects, ‘u’ is a vector with random effects (kinship matrix), ‘e’ is a residuals vector, X denotes the accessions/genotypes at the marker, ‘Q’ is the Q-matrix, the result of ADMIXTURE software, and ‘Z’ is an identity matrix.

The GLM principally considers the population structure (Q) while MLM considers both Q and Kinship (K). Further, among the different options available within MLM, the widely adapted approach called ‘optimum levels of compression in combination with P3D’ for variance component estimation was used for association analysis. For the MLM analysis, marker-based kinship matrix (K) obtained using TASSEL was used along with the Q matrix generated through ADMIXTURE to correct for both family and population structure and the phenotypic variation explained (R^2^) by the marker is reported^74,77^. Quantile–Quantile (Q-Q) plots were developed by plotting observed negative Log_10_ ‘*P’* values against expected negative Log_10_ ‘*P*’ values for all the available SNPs in R package CMplot^78^. A deviation from ‘*P*’ values at the initial stage may display the existing population stratification. Manhattan plots were used to visualize chromosome-wise SNPs obtained through the marker-trait association study performed across the genome. -Log_10_ of the ‘*P*’ value for each SNP was plotted against seven chromosomes for the respective trait. Based on the SNP distribution, the threshold for significance of associations between SNPs and traits was fixed at [− log 10 (p) < 10^−03^] which gave the optimum number of reliable SNPs. SNP density plots, Q-Q plots, and Manhattan plots were generated using R package CMplot v 3.4.0^78^.

The corresponding genes of associated SNPs or marker-trait associations were identified by using the physical positions of SNPs in gene annotations available in the pearl millet reference genome sequence^2^; and thus the functions of the respective SNPs were determined.

### Candidate genes discovery

The candidate genes corresponding to the significantly associated SNPs were identified using the pearl millet genome^2^ sequence annotations. The SNP subsiding start and end positions of a gene or exons were explored for candidate genes based on their biological function annotation related to the trait of interest (Supplementary Fig. S2). It is possible to obtain multiple SNPs on a gene segment which are referred to as haplotypes^79^.

## Supplementary information

Supplementary Information.
