# Supplementary material for: Genome-wide association study uncovers genomic regions associated with grain iron, zinc and protein content in pearl millet
Source: Sci Rep. 2020 Nov 10;10:19473. doi: 10.1038/s41598-020-76230-y (PMC7655845; doi:10.1038/s41598-020-76230-y)
Supplement: Supplementary file 1 — Supplementary Information. [file 41598_2020_76230_MOESM1_ESM.docx]

**Supplementary Information**

**(Supplementary Tables and Figures)**

**Genome-wide association study uncovers genomic regions associated with grain iron, zinc, and protein content in pearl millet**

**Mahesh Pujar^1,2^, S. Gangaprasad^2^, Mahalingam Govindaraj^1*^, Sunil S. Gangurde^1^_,_ A Kanatti^1^, Himabindu Kudapa^1^**

^1^ International Crops Research Institute for the Semi-Arid Tropics (ICRISAT), Patancheru-502 324, Telangana, India;

^2^ University of Agricultural Sciences Shivamogga-577 225, Karnataka, India:

* Correspondence: [m.govindaraj@cgiar.org](mailto:m.govindaraj@cgiar.org); Ph. No: +91 4030 713617

Table S1. Chromosome wise marker trait associations (MTAs) identified through genome wide association study (GWAS) for the iron (Fe), zinc (Zn) and protein content (PC)

| **Traits** | **Total SNPs per traits** | **SNPs per chromosome** | **Chromosome Number** | **SNP markers** |
| --- | --- | --- | --- | --- |
| Fe | 18 | 1 | Pgl01 | Pgl01_157344213 |
|  |  | 4 | Pgl02 | Pgl02_8191, Pgl02_64976379, Pgl02_69249845, Pgl02_233052877 |
|  |  | 7 | Pgl04 | Pgl04_190105720, Pgl04_15506741, Pgl04_17259669, Pgl04_23381732, Pgl04_32057582, Pgl04_32617883, **Pgl04_64673688** |
|  |  | 3 | Pgl05 | Pgl05_107148808, **Pgl05_135500493, Pgl05_144482656** |
|  |  | 2 | Pgl06 | Pgl06_21219367,Pgl06_145237122 |
|  |  | 1 | Pgl07 | **Pgl07_101483782** |
| Zn | 43 | 5 | Pgl01 | Pgl01_568786, Pgl01_51414126, Pgl01_172878523, Pgl01_177992632, Pgl01_218681895 |
|  |  | 1 | Pgl02 | Pgl02_69256531 |
|  |  | 3 | Pgl03 | Pgl03_180499360, Pgl03_4732348, Pgl03_13329915 |
|  |  | 6 | Pgl04 | Pgl04_1518626, Pgl04_9044259, Pgl04_9059217, Pgl04_64429980, **Pgl04_64673688**, Pgl04_74518920 |
|  |  | 12 | Pgl05 | Pgl05_85608777, Pgl05_91509511, Pgl05_92617645, Pgl05_92926570, Pgl05_98096070, Pgl05_104608199, **Pgl05_135500493**, Pgl05_143124835, Pgl05_143702980, Pgl05_143706557, **Pgl05_144482656**, Pgl05_148964458 |
|  |  | 5 | Pgl06 | Pgl06_223926259, Pgl06_231796045, Pgl06_18558795, Pgl06_36628895, Pgl06_54978917 |
|  |  | 11 | Pgl07 | Pgl07_9399240, Pgl07_19060446 Pgl07_19133990, Pgl07_20613468, Pgl07_35376984, **Pgl07_101483782**, Pgl07_101483780, Pgl07_101517680, Pgl07_125865145, Pgl07_147179490, Pgl07_151365061 |
| PC | 17 | 7 | Pgl01 | Pgl01_44640725, Pgl01_44640726, Pgl01_177992633, Pgl01_177992634, Pgl01_250761833 Pgl01_266542617, Pgl01_266542615 |
|  |  | 5 | Pgl02 | Pgl02_28323518, Pgl02_182371002, Pgl02_225493497, Pgl02_225493495, Pgl02_241839676 |
|  |  | 1 | Pgl04 | Pgl04_32176024 |
|  |  | 1 | Pgl05 | Pgl05_156574366 |
|  |  | 1 | Pgl06 | Pgl06_71295563 |
|  |  | 2 | Pgl07 | Pgl07_124769335, Pgl07_124769336 |

Table S2. Common SNPs identified across iron (Fe) and zinc (Zn)

| **Marker-ID** | **Chromosome Numbers** | **Common Traits** | **P value range** | **R^2^/PVE** |
| --- | --- | --- | --- | --- |
| Pgl04_64673688 | Pgl04 | Fe and Zn | 2.53E-04 to 4.23E-04 | 5.7 to 6.1 |
| Pgl05_135500493 | Pgl05 | Fe and Zn | 1.79E-05 to 1.72E-04 | 6.43 to 8.23 |
| Pgl05_144482656 | Pgl05 | Fe and Zn | 9.03E-05 to 8.82E-04 | 5.18 to 6.88 |
| Pgl07_101483782 | Pgl07 | Fe and Zn | 3.22E-04 to 2.24E-05 | 5.91 to 8.0 |

Table S3: Gene annotation for 78 marker trait associations (MTAs) identified using genome wide association study

| Trait | Marker | Locus position | GO annotation | Annotations |
| --- | --- | --- | --- | --- |
| Fe | Pgl01_157344213 | 4945 | - | IPR001163; Like-Sm ribonucleoprotein (LSM) domain\|IPR006649; Like-Sm ribonucleoprotein (LSM) domain, eukaryotic/archaea-type |
| Fe | Pgl02_8191 | 10405 | GO:0003700; sequence-specific DNA binding transcription factor activity; Molecular Function\|GO:0006355; regulation of transcription, DNA-dependent; Biological Process\|GO:0043565; sequence-specific DNA binding; Molecular Function\|GO:0046983; protein dimerization activity; Molecular Function | IPR004827; Basic-leucine zipper domain\|IPR011616; bZIP transcription factor, bZIP-1 |
| Fe | Pgl02_64976379 | 14868 | GO:0003677; DNA binding; Molecular Function\|GO:0005515; protein binding; Molecular Function\|GO:0005634; nucleus; Cellular Component | IPR000048; IQ motif, EF-hand binding site\|IPR002110; Ankyrin repeat\|IPR002909; Cell surface receptor IPT/TIG\|IPR005559; CG-1 DNA-binding domain\|IPR020683; Ankyrin repeat-containing domain |
| Fe | Pgl02_69249845 | 15083 | GO:0009790; embryo development; Biological Process | IPR005513; Late embryogenesis abundant protein, LEA-25/LEA-D113 |
| Fe | Pgl02_233052877 | 20238 | GO:0005515; protein binding; Molecular Function | IPR001611; Leucine-rich repeat\|IPR003591; Leucine-rich repeat, typical subtype\|IPR013210; Leucine-rich repeat-containing N-terminal, type 2 |
| Fe | Pgl04_190105720 | 31373 | GO:0005515; protein binding; Molecular Function\|GO:0008270; zinc ion binding; Molecular Function | IPR001841; Zinc finger, RING-type |
| Fe | Pgl04_15506741 | 32743 | - | - |
| Fe | Pgl04_17259669 | 32839 | - | - |
| Fe | Pgl04_23381732 | 33229 | GO:0016881; acid-amino acid ligase activity; Molecular Function | IPR000608; Ubiquitin-conjugating enzyme, E2 |
| Fe | Pgl04_32057582 | 33609 | - | - |
| Fe | Pgl04_32617883 | 33639 | - | IPR004314; Domain of unknown function DUF239\|IPR025521; Domain of unknown function DUF4409 |
|  |  |  |  |  |
| Fe | Pgl04_64673688 | 35337 | - | - |
| Fe | Pgl05_107148808 | 42149 | GO:0005506; iron ion binding; Molecular Function\|GO:0009055; electron carrier activity; Molecular Function\|GO:0016705; oxidoreductase activity, acting on paired donors, with incorporation or reduction of molecular oxygen; Molecular Function\|GO:0020037; heme binding; Molecular Function\|GO:0055114; oxidation-reduction process; Biological Process | IPR001128; Cytochrome P450\|IPR002401; Cytochrome P450, E-class, group I |
| Fe | Pgl05_135500493 | 43652 | - | - |
| Fe | Pgl05_144482656 | 44420 | - | - |
| Fe | Pgl06_21219367 | 49082 | GO:0005215; transporter activity; Molecular Function\|GO:0006857; oligopeptide transport; Biological Process\|GO:0016020; membrane; Cellular Component | IPR000109; Oligopeptide transporter\|IPR018456; PTR2 family proton/oligopeptide symporter, conserved site |
| Fe | Pgl06_145237122 | 52081 | - | - |
| Fe | Pgl07_101483782 | 56785 | - | IPR002885; Pentatricopeptide repeat |
| Zn | Pgl01_568786 | 35 | GO:0005524; ATP binding; Molecular Function | IPR001023; Heat shock protein Hsp70\|IPR013126; Heat shock protein 70\|IPR018181; Heat shock protein 70, conserved site |
| Zn | Pgl01_51414126 | 2455 | GO:0004672; protein kinase activity; Molecular Function\|GO:0004674; protein serine/threonine kinase activity; Molecular Function\|GO:0004713; protein tyrosine kinase activity; Molecular Function\|GO:0005515; protein binding; Molecular Function\|GO:0005524; ATP binding; Molecular Function\|GO:0006468; protein phosphorylation; Biological Process | IPR000719; Protein kinase, catalytic domain\|IPR001245; Serine-threonine/tyrosine-protein kinase catalytic domain\|IPR001611; Leucine-rich repeat\|IPR002290; Serine/threonine- / dual-specificity protein kinase, catalytic domain\|IPR003591; Leucine-rich repeat, typical subtype\|IPR008271; Serine/threonine-protein kinase, active site\|IPR013210; Leucine-rich repeat-containing N-terminal, type 2\|IPR017441; Protein kinase, ATP binding site\|IPR020635; Tyrosine-protein kinase, catalytic domain |
| Zn | Pgl01_172878523 | 5411 | - | - |
| Zn | Pgl01_177992632 | 5631 | GO:0004672; protein kinase activity; Molecular Function\|GO:0004713; protein tyrosine kinase activity; Molecular Function\|GO:0005515; protein binding; Molecular Function\|GO:0005524; ATP binding; Molecular Function\|GO:0006468; protein phosphorylation; Biological Process | IPR000719; Protein kinase, catalytic domain\|IPR001245; Serine-threonine/tyrosine-protein kinase catalytic domain\|IPR001611; Leucine-rich repeat\|IPR002290; Serine/threonine- / dual-specificity protein kinase, catalytic domain\|IPR013210; Leucine-rich repeat-containing N-terminal, type 2\|IPR020635; Tyrosine-protein kinase, catalytic domain |
| Zn | Pgl01_218681895 | 6772 | GO:0004176; ATP-dependent peptidase activity; Molecular Function\|GO:0006508; proteolysis; Biological Process | IPR003111; Peptidase S16, lon N-terminal |
| Zn | Pgl02_69256531 | 15090 | GO:0003677; DNA binding; Molecular Function | IPR001005; SANT/Myb domain\|IPR015495; Myb transcription factor\|IPR017930; Myb domain, DNA-binding |
| Zn | Pgl03_180499360 | 21664 | - | - |
| Zn | Pgl03_4732348 | 26569 | - | - |
| Zn | Pgl03_13329915 | 26934 | - | - |
| Zn | Pgl04_1518626 | 31638 | GO:0006952; defense response; Biological Process\|GO:0043531; ADP binding; Molecular Function | IPR000767; Disease resistance protein\|IPR002182; NB-ARC |
| Zn | Pgl04_9044259 | 32317 | - | - |
| Zn | Pgl04_9059217 | 32322 | - | - |
| Zn | Pgl04_64429980 | 35310 | GO:0005515; protein binding; Molecular Function | IPR000210; BTB/POZ-like\|IPR002083; MATH\|IPR013069; BTB/POZ |
| Zn | Pgl04_64673688 | 35337 | - | - |
| Zn | Pgl04_74518920 | 35844 | GO:0005515; protein binding; Molecular Function | IPR002083; MATH |
| Zn | Pgl05_85608777 | 40955 | GO:0016757; transferase activity, transferring glycosyl groups; Molecular Function | IPR002495; Glycosyl transferase, family 8 |
| Zn | Pgl05_91509511 | 41274 | GO:0005215; transporter activity; Molecular Function\|GO:0006857; oligopeptide transport; Biological Process\|GO:0016020; membrane; Cellular Component | IPR000109; Oligopeptide transporter\|IPR018456; PTR2 family proton/oligopeptide symporter, conserved site |
| Zn | Pgl05_92617645 | 41322 | - | - |
| Zn | Pgl05_92926570 | 41352 | - | IPR019141; Protein of unknown function DUF2045 |
| Zn | Pgl05_98096070 | 41649 | - | IPR008546; Domain of unknown function DUF828\|IPR013666; Pleckstrin-like, plant |
| Zn | Pgl05_104608199 | 42014 | - | IPR011676; Domain of unknown function DUF1618 |
| Zn | Pgl05_135500493 | 43652 | GO:0009058; biosynthetic process; Biological Process | IPR001296; Glycosyl transferase, family 1 |
| Zn | Pgl05_143124835 | 44259 | GO:0003677; DNA binding; Molecular Function\|GO:0006306; DNA methylation; Biological Process | IPR001525; C-5 cytosine methyltransferase\|IPR015940; Ubiquitin-associated/translation elongation factor EF1B, N-terminal, eukaryote |
| Zn | Pgl05_143702980 | 44334 | GO:0003677; DNA binding; Molecular Function\|GO:0005524; ATP binding; Molecular Function\|GO:0005634; nucleus; Cellular Component\|GO:0006260; DNA replication; Biological Process\|GO:0006270; DNA-dependent DNA replication initiation; Biological Process | IPR001208; Mini-chromosome maintenance, DNA-dependent ATPase\|IPR008045; Mini-chromosome maintenance complex protein 2\|IPR018525; Mini-chromosome maintenance, conserved site |
| Zn | Pgl05_143706557 | 44339 | GO:0003677; DNA binding; Molecular Function\|GO:0005524; ATP binding; Molecular Function\|GO:0005634; nucleus; Cellular Component\|GO:0006260; DNA replication; Biological Process\|GO:0006270; DNA-dependent DNA replication initiation; Biological Process | IPR001208; Mini-chromosome maintenance, DNA-dependent ATPase\|IPR008045; Mini-chromosome maintenance complex protein 2\|IPR018525; Mini-chromosome maintenance, conserved site |
| Zn | Pgl05_144482656 | 44420 | - | - |
| Zn | Pgl05_148964458 | 44843 | GO:0005622; intracellular; Cellular Component\|GO:0042254; ribosome biogenesis; Biological Process | IPR001790; Ribosomal protein L10/acidic P0 |
| Zn | Pgl06_223926259 | 46965 | - | - |
| Zn | Pgl06_231796045 | 47458 | GO:0003677; DNA binding; Molecular Function | IPR001005; SANT/Myb domain\|IPR017930; Myb domain, DNA-binding |
| Zn | Pgl06_18558795 | 48867 | GO:0008168; methyltransferase activity; Molecular Function\|GO:0031167; rRNA methylation; Biological Process | IPR004398; RNA methyltransferase, RsmD |
| Zn | Pgl06_36628895 | 49710 | - | - |
| Zn | Pgl06_54978917 | 50131 | GO:0005737; cytoplasm; Cellular Component\|GO:0006139; nucleobase-containing compound metabolic process; Biological Process\|GO:0006281; DNA repair; Biological Process\|GO:0006310; DNA recombination; Biological Process\|GO:0006974; response to DNA damage stimulus; Biological Process\|GO:0016788; hydrolase activity, acting on ester bonds; Molecular Function | IPR005227; Resolvase, holliday junction-type, YqgF-like\|IPR006641; YqgF/RNase H-like domain |
| Zn | Pgl07_9399240 | 53210 | - | IPR004182; GRAM |
| Zn | Pgl07_19060446 | 53754 | GO:0005507; copper ion binding; Molecular Function\|GO:0009055; electron carrier activity; Molecular Function | IPR000923; Blue (type 1) copper domain\|IPR003245; Plastocyanin-like |
| Zn | Pgl07_19133990 | 53759 | GO:0004252; serine-type endopeptidase activity; Molecular Function\|GO:0006508; proteolysis; Biological Process | IPR000209; Peptidase S8/S53, subtilisin/kexin/sedolisin\|IPR015500; Peptidase S8, subtilisin-related\|IPR022398; Peptidase S8, subtilisin, His-active site |
| Zn | Pgl07_20613468 | 53826 | - | - |
| Zn | Pgl07_35376984 | 54404 | GO:0016620; oxidoreductase activity, acting on the aldehyde or oxo group of donors, NAD or NADP as acceptor; Molecular Function\|GO:0055114; oxidation-reduction process; Biological Process | IPR004262; Male sterility\|IPR013120; Male sterility, NAD-binding |
| Zn | Pgl07_101483782 | 56785 | - | IPR002885; Pentatricopeptide repeat |
| Zn | Pgl07_101483780 | 56786 | - | IPR002885; Pentatricopeptide repeat |
| Zn | Pgl07_101517680 | 56792 | GO:0016746; transferase activity, transferring acyl groups; Molecular Function | IPR012328; Chalcone/stilbene synthase, C-terminal |
| Zn | Pgl07_125865145 | 57755 | GO:0006952; defense response; Biological Process\|GO:0043531; ADP binding; Molecular Function | IPR000767; Disease resistance protein\|IPR002182; NB-ARC |
| Zn | Pgl07_147179490 | 58482 | - | - |
| Zn | Pgl07_151365061 | 58621 | - | - |
| PC | Pgl01_44640725 | 2139 | - | - |
| PC | Pgl01_44640726 | 2140 | GO:0005515; protein binding; Molecular Function | IPR002035; von Willebrand factor, type A |
| PC | Pgl01_266542615 | 9243 | GO:0005515; protein binding; Molecular Function | IPR002035; von Willebrand factor, type A |
| PC | Pgl01_177992633 | 5632 | GO:0003700; sequence-specific DNA binding transcription factor activity; Molecular Function\|GO:0006355; regulation of transcription, DNA-dependent; Biological Process\|GO:0043565; sequence-specific DNA binding; Molecular Function | IPR001356; Homeodomain\|IPR002913; Lipid-binding START\|IPR013978; MEKHLA |
| PC | Pgl01_177992634 | 5633 | GO:0004672; protein kinase activity; Molecular Function\|GO:0004713; protein tyrosine kinase activity; Molecular Function\|GO:0005515; protein binding; Molecular Function\|GO:0005524; ATP binding; Molecular Function\|GO:0006468; protein phosphorylation; Biological Process | IPR000719; Protein kinase, catalytic domain\|IPR001245; Serine-threonine/tyrosine-protein kinase catalytic domain\|IPR001611; Leucine-rich repeat\|IPR002290; Serine/threonine- / dual-specificity protein kinase, catalytic domain\|IPR013210; Leucine-rich repeat-containing N-terminal, type 2\|IPR020635; Tyrosine-protein kinase, catalytic domain |
| PC | Pgl01_250761833 | 7975 | GO:0004672; protein kinase activity; Molecular Function\|GO:0004674; protein serine/threonine kinase activity; Molecular Function\|GO:0004713; protein tyrosine kinase activity; Molecular Function\|GO:0005524; ATP binding; Molecular Function\|GO:0006468; protein phosphorylation; Biological Process\|GO:0006486; protein glycosylation; Biological Process\|GO:0008373; sialyltransferase activity; Molecular Function\|GO:0030173; integral to Golgi membrane; Cellular Component | IPR000719; Protein kinase, catalytic domain\|IPR001245; Serine-threonine/tyrosine-protein kinase catalytic domain\|IPR001675; Glycosyl transferase, family 29\|IPR002290; Serine/threonine- / dual-specificity protein kinase, catalytic domain\|IPR008271; Serine/threonine-protein kinase, active site\|IPR017441; Protein kinase, ATP binding site\|IPR020635; Tyrosine-protein kinase, catalytic domain |
| PC | Pgl01_266542617 | 9242 | - | - |
| PC | Pgl01_266542615 | 9243 | GO:0005515; protein binding; Molecular Function | IPR002035; von Willebrand factor, type A |
| PC | Pgl02_28323518 | 12647 |  |  |
| PC | Pgl02_182371002 | 18163 | - | - |
| PC | Pgl02_225493497 | 19831 | - | - |
| PC | Pgl02_225493495 | 19832 | - | - |
| PC | Pgl02_241839676 | 21108 | GO:0005506; iron ion binding; Molecular Function\|GO:0009055; electron carrier activity; Molecular Function\|GO:0016705; oxidoreductase activity, acting on paired donors, with incorporation or reduction of molecular oxygen; Molecular Function\|GO:0020037; heme binding; Molecular Function\|GO:0055114; oxidation-reduction process; Biological Process | IPR001128; Cytochrome P450 |
| PC | Pgl05_156574366 | 38205 | - | - |
| PC | Pgl06_71295563 | 50682 | - | - |
| PC | Pgl07_124769335 | 57696 |  |  |
| PC | Pgl07_124769336 | 57697 |  | IPR006869; Domain of unknown function DUF547 |

Table S4. 281 inbred lines and their pedigree information used for genome wide association study (GWAS).

| **Treatment No** | **Inbred line Designation** | **Pedigree** |
| --- | --- | --- |
| 1 | ICMB 100646 | (EEBC S1-407-1-B-B-B-B-B-1-B-1-B-10-1 x B-bulk (3981-3989/S06 G1))-1-2-1 |
| 2 | ICMB 100485 | {[(843B x ICTP 8202-161-5)-20-3-B-B-3 x B-bulk]-2-B-9 x [(ICMB 96555 x LaGrap C2 S1-32-1)-10 x IP 14758-2-1]-8-2}-1-1-1-2 |
| 3 | ICMB 100648 | {[(BESCBPT/91-40 x SPF3/S91-3)-1-2-2-3 x B-bulk]-8-1-1-3-B-B-B-B-3-1 x B-bulk (3981-4011/S06 G1)}-1-3-2-1-3 |
| 4 | ICMB 100649 | {EEBC S1-407-1-B-B-B-B-B-1-B-1-B-5-1x 3981-3989 G1}-2-1-3 |
| 5 | ICMB 100650 | EEDBC S1-2-1-1-1-1-1-1-B-B-1-4-B-B-4 |
| 6 | ICMB 100651 | {(MC 94 S1-34-1-B x HHVBC)-16-2-1-1-1-1-B-B-5 x (MC 94 S1-34-1-B x HHVBC)-10-4-1-2-1-B-B-1-30-2-4-2-1-3-4-3 |
| 7 | ICMB 100219 | (EEBC S1-407-1-B-B-B-B-B-1-B-1-B-10-1 x B-bulk (3981-3989/S06 G1))-1-1-1 |
| 8 | ICMB 100654 | (EEBC S1-407-1-B-B-B-B-B-1-B-1-B-10-1 x B-bulk (3981-3989/S06 G1))-1-2-4-B |
| 9 | ICMB 100454 | (EEBC S1-407-1-B-B-B-B-B-1-B-1-B-5-1 x B-bulk (3981-3989/S06 G1))-2-1-3 |
| 10 | ICMB 100409 | (EEDBC S1-465-2-2-2-B-B-2-B-8-1 x B-bulk (3981-4011/S06 G1))-2-1-3-BxEarly D2/Med group lines x med maturity lines bulk (1301-281)-8-3-1 |
| 11 | ICMB 100655 | (ICMB 02111 x (ICMB 96555 x ICMB 99111)-2-1-2-3)-9-5-1 |
| 12 | ICMP 100409 | IP 12181 S1-1-1-1-2-2 |
| 13 | ICMB 100656 | (ICMB 04888 x ICMB 02333)-3-1-2-3-2 |
| 14 | ICMB 100657 | (ICMB 96555 x IP 10437)-3-4-1-2-8 x {(96555B x LaGrap C2 S1-32-1)-10}xIP 14758-2-1]-4-2-1-5-3-1-1 |
| 15 | ICMB 100465 | (NC D2 BC7F4-34-3-1-2-B-2-B x EEBC 407)-12-1-2 |
| 16 | ICMB 100658 | [(ICMB 95555 x ICMB 94333)-8-2-1-B-B-6-1 x B-bulk (3981-4011/S06 G1)]-2-5-4-BxEarly D2/Med group lines x med maturity lines bulk (1301-28)-3-3-1-1 |
| 17 | ICMB 100025 | [(ICMB 95555 x ICMB 94333)-8-2-1-B-B-9-1 x B-bulk (3981-4011/S06 G1)]-2-3-4-B |
| 18 | ICMB 100659 | [(ICMR 312 S1-1-5-2-B x HHVBC)-10-2-1-2-3 x EEBC 407)-7-2-1 |
| 19 | ICMB 100660 | [(MC 94 S1-34-1-B x HHVBC)-16-1-3-1-2-2-B-B-2-B-B x ICMB 00444]-14-5-1-1xEarly D2/Med group lines x med maturity lines bulk (1301-28)-3-2-1 |
| 20 | ICMB 100480 | [{(81B x 4017-6-1-1)-3-1-4-3-4-1-2-1-B-2-2 x ICMB 05888}x (HHVDBC Medium HS-120-1-2-1-1-1-1 x HHVDBC Medium HS-15-1-1-1-2-2-4)]-19-2-2-4 |
| 21 | ICMB 100661 | [{(ICMB 96555 x IP 10437)-9-B-B-B-B-B-B x IP 14758-2-2}-19-1-B x (ICMB 96555 x IP 10437)-3-4-1-2-2-1-B-2-B-3]-1-1-1-1-3 |
| 22 | ICMB 100075 | [{(ICMB 96555 x IP 10437)-9-B-B-B-B-B-B x IP 14758-2-2}-19-1-B x (ICMB 96555 x IP 10437)-3-4-1-2-2-1-B-2-B-3]-3-3-2-1-5 |
| 23 | ICMB 100662 | [{(SRC II C3 S1-1-3-2 x HHVBC)-9-5-3-1-B P1-3-B-3-B x ICMB 05888} x (HHVDBC Medium HS-120-1-2-1-1-1-1 x HHVDBC Medium HS-15-1-1-1-2-2-4)]-16-1-2-2 |
| 24 | ICMB 100663 | [EEDBC S1-452-3-1-2-3-B-B-B-1 x B-bulk (3981-3989/S06 G1)]-4-1-3 |
| 25 | ICMB 100664 | [ICMB 95111 x (D2BLN/95-107 x EEBC C1-1)-6-B]-24-4-1-B-B-B-B-11-1] x B-bulk (3981-3989/S06 G1)}-2-3-1-B |
| 26 | ICMB 100665 | {(EEDBC S1-425-2-1-2-3-B-2-2-4 x NCD2S1-20-7-2-4-1-4-2-3) x (HHVDBC Medium HS-120-1-2-1-1-1-1 x HHVDBC Medium HS-15-1-1-1-2-2-4)}-9-1-4-4-2 |
| 27 | ICMB 100666 | {(ICMB 96555 x IP 10437)-3-4-1-2-8 x [(ICMB 96555 x LaGrap C2 S1-32-1)-10 x IP 14758-2-1]}-8-3-2-4-1-2 |
| 28 | ICMB 100667 | {[(843B x ICTP 8202-161-5)-20-3-B-B-3 x B-bulk]-2-B-1-2-2-B-B-B-11-1 x B-bulk (3981-4011/S06 G1)}-3-2-4-1xEarly D2/Med group lines x med maturity lines bulk (1301-28)-15-1-2-1 |
| 29 | ICMB 100668 | {[(843B x ICTP 8202-161-5)-20-3-B-B-3 x B-bulk]-2-B-1-2-2-B-B-B-11-1 x B-bulk (3981-4011/S06 G1)}-3-2-4-4-1-2 |
| 30 | ICMB 100669 | {[(MC 94 S1-81-1-B x HHVBC)-4-4-1 x (MC 94 S1-81-1-B x HHVBC)-4-2-4-7-1-1-B-2] x (ICMR 312 S1-1-5-2-B x HHVBC)-10-2-1-2}-8-2-3-1 |
| 31 | ICMB 1503 | {[D2BLN/95-312 x (96333B x HHVBC)-1-B-1-B-B-2-B-B-B-5-1] x B-bulk (3981-4011/S06 G1)}-3-1-1 |
| 32 | ICMB 100455 | EEDBC S1-2-1-1-1-1-1-1-B-B-1-4-B-B |
| 33 | ICMB 100081 | HHVDBC HS-120-1-2-1-1-3-B-4 |
| 34 | ICMB 100670 | IC-CZBC-C0-60-4-4-2-B-B-B-1 |
| 35 | ICMB 100672 | (ICMB 02444 x ICMB 00888)-19-1-B-1-6-2-B-B |
| 36 | ICMB 100673 | (ICMB 00888 x ICMB 03999)-26 -5-1-3-B-3-B |
| 37 | ICMB 100411 | (ICMB 04999 x ICMB 02444)-2 -4-2-6-B-1-1 |
| 38 | ICMB 100235 | (ICMB 99444xICMB 99222)-1-4-2-1-4-1-1-B |
| 39 | ICMB 100304 | (MC 94 C2-S1-3-2-2-2-1-3-B-B x ICMR 312 S1-3-2-3-2-1-1-B-B)-B-34-4-1-d2-3 |
| 40 | ICMB 100305 | [(ICMR 312 S1-1-5-2-B x HHVBC)-10-2-1-2-3-B x HHVDBC HS-158-2-1-2-1-1-B]-9-2-3-1 |
| 41 | ICMB 100674 | (ICMB 04888 x ICMB 02444)-1-4-B-1-2-2-1 |
| 42 | ICMB 100675 | ([78-7088/3/SER3 AD//B282/(3/4)EB x PBLN/S95-359]-7-4-B-B-2-B-B x (EEBC S1-407-1-B-B-B-B-B-1-B-1-B-13-1 x B-bulk (3981-3989/S06 G1))-1-2-3)-4-1-3 |
| 43 | ICMB 100677 | [(EEBC S1-407-1-B-B-B-B-B-1-B-1-B-5-1x 3981-3989 G1)-2-1-1x(ICMB 04888 x ICMB 02333)-3-1-3-1]-1-1-2-1-3 |
| 44 | ICMB 100678 | [(EEBC S1-407-1-B-B-B-B-B-1-B-1-B-5-1x 3981-3989 G1)-2-1-1x{[(843B x ICTP 8202-161-5)-20-3-B-B-3 x B-bulk]-2-B-9 x [(ICMB 96555 x LaGrap C2 S1-32-1)-10 x IP 14758-2-1]-8-2}-1-1-2-2]-2-1-2-1-3-1 |
| 45 | ICMB 100679 | [(EEBC S1-407-1-B-B-B-B-B-1-B-1-B-13-1 x B-bulk (3981-3989/S06 G1))-1-2-3x[(ICMR 312 S1-1-5-2-B x HHVBC)-10-2-1-2-3 x EEBC 407)-7-2-1-3]-24-5-1-1-3-1 |
| 46 | ICMB 100680 | [EEDBC S1-425-2-1-2-3-B-1-B-8-1x 3981-4011 G2}-3-1-3x(EEBC S1-407-1-B-B-B-B-B-1-B-1-B-5-1 x B-bulk (3981-3989/S06 G1))-2-1-3]-11-1-2-3-1 |
| 47 | ICMB 100248 | [(EEBC S1-407-1-B-B-B-B-B-1-B-1-B-5-1 x B-bulk (3981-3989/S06 G1))-2-1-3 X AIMP 92901-S1-278-1-1]-46-5-1-3 |
| 48 | ICMB 100252 | (ARD-288-1-10-1-2 (RM)-5-P2-B x 40258/S14-P7)-20-P1 |
| 49 | ICMP 100445 | ICTP 8203 S1-166-3-4-2-1 |
| 50 | ICMB 02333 | (BSECBPT/91-39 x SPF3/S91-116)-15-2-1-2 |
| 51 | ICMB 99222 | (BSECBPT/91-40 x SPF3/S91-94)-3-1-1-2 |
| 52 | ICMR 100973 | (EERC-HS-24)-B-2-3-2-1-B |
| 53 | ICMR 100974 | (IPC 337 ×SDMV 90031-S1-84-1-1-1-1)-2-1-5-P1-1 |
| 54 | ICMR 13777 | (MC 94 C2-S1-3-1-3-3-1-2-1 x SDMV 90031 S1-3-3-2-2-2-2-2)-B-8-2-1 |
| 55 | ICMR 100327 | (MC 94 C2-S1-3-2-2-2-1-3-B-B x ICMR 312 S1-3-2-3-2-1-1-B-B)-B-34-4-1-2 |
| 56 | ICMR 100300 | [(843B x ICTP 8202-161-5)-20-3-B-B-3 x B-lines bulk]-2-B-9x{(96555B x LaGrap C2 S1-32-1)-10}xIP 14758-2-1]-8-2-1-1-1 |
| 57 | ICMR 06555 | AIMP 92901 S1-296-2-1-1-3-B-1-B-B |
| 58 | ICMR 100725 | AIMP 92901 S1-296-2-1-1-4-2-B-7-3-1 |
| 59 | ICMR 100975 | ICMR 312 S1-3-2-1-2-2-B-2-1-1 |
| 60 | ICMR 100444 | ICMR 312 S1-3-2-1-2-4-1 |
| 61 | ICMR 100976 | ICMV 96490-S1-15-1-2-1-1-B |
| 62 | ICMR 100977 | ICMV 96490-S1-15-1-2-1-3-1-B |
| 63 | ICMR 100978 | IP No. 17580-1-B-B-B-B-B-B-B-1 |
| 64 | ICMR 100979 | LaGrap C2-S1-14-1-2-3-3-2-B |
| 65 | ICMR 100920 | SDMV 90031-S1-3-3-2-1-3-2-2-3-2 |
| 66 | ICMR 100299 | (EERC-HS-34)-B-7-2-3-2 |
| 67 | ICMP 100239 | (EERC-HS-8)-27-1 |
| 68 | ICMR 100981 | (MC 94 C2-S1-3-1-3-3-1-2-1 x SDMV 90031 S1-3-3-2-2-2-2-2)-B-8-2-1-2 |
| 69 | ICMR 100982 | (MC 94 C2-S1-3-2-2-2-1-3-B-B x AIMP 92901 S1-488-2-1-1-4-B-B)-B-30-3-1-6-2x R-lines bulk (20216-20249/K09)] |
| 70 | ICMR 100983 | (MC 94 C2-S1-3-2-2-2-1-3-B-B x ICMR 312 S1-3-2-3-2-1-1-B-B)-B-7-2-1-B-B-B-B |
| 71 | ICMR 100984 | [(AIMP 92901 S1-488-2-1-1-2-B-1-B x R-lines bulk (20216-20249/K09)]-6-3-1 |
| 72 | ICMR 100985 | [(AIMP 92901 S1-488-2-1-1-2-B-1-B x R-lines bulk (20216-20249/K09)]-8-1-3 |
| 73 | ICMR 100445 | [(ICTP 8202 S1-99-2 x R-lines bulk (20216-20249/K09)]-12-1 |
| 74 | ICMR 100986 | [(IPC 1617×SDMV 90031-S1-84-1-1-1-1)×AIMP 92901 S1-296-2-1-1-3-B-1]-3-3-1-1-B |
| 75 | ICMR 100987 | [(IPC 1617×SDMV 90031-S1-84-1-1-1-1)×AIMP 92901 S1-296-2-1-1-3-B-1]-4-4-2-3-3-1 |
| 76 | ICMR 100988 | [ICMR 312 S1-3-2-1-2-4 x LaGrap C2-S1-64]-B-27-2-1 |
| 77 | ICMR 100019 | AIMP 92901 S1-296-2-1-1-3-B-3-2-B-2-B-B |
| 78 | ICMR 100989 | AIMP 92901 S1-296-2-1-1-3-B-3-2-B-2-B-B-19-2x R-lines bulk (20216-20249/K09)]-1-3 |
| 79 | ICMR 100991 | ICMR 312 S1-8-1-1-1-1-B-B-B-1-B-22-3x R-lines bulk (20216-20249/K09)]-2 |
| 80 | ICMR 100992 | ICMV 96490-S1-15-1-2-1-3-2-B-B |
| 81 | ICMR 100028 | Jakhrana × ESRC II S2-11-B-1-2-1-1-B |
| 82 | ICMR 100993 | (EERC-HS-7)-9-2-1 |
| 83 | ICMR 100994 | {[[(MC 94 C2-S1-3-2-2-2-1-3-B-B x ICMR 312 S1-3-2-3-2-1-1-B-B)]-B-30-3-1x R-lines bulk (20216-20249/K09)} -9-1 |
| 84 | ICMR 100056 | (([(IPC 1617×SDMV 90031-S1-84-1-1-1-1)×AIMP 92901 S1-296-2-1-1-3-B-1]-4-4-5-3-2) X (LaGrap C2-S1-38-2-1-1-1)-6-1-B |
| 85 | ICMR 100038 | [[(IPC 1617×SDMV 90031-S1-84-1-1-1-1)×AIMP 92901 S1-296-2-1-1-3-B-1]-4-4-2-1 X ICMV 96490-S1-15-4-1-1-2]-29-1-1 |
| 86 | ICMR 100040 | [AIMP 92901 S1-296-2-1-1-4-2-B-15-2-1 X ICMV 96490-S1-15-1-2-3-2]-30-1-1 |
| 87 | ICMR 100098 | (GGP S1-79-1XAIMP 92901-S1-10-1-3)-4-3-2 |
| 88 | ICMR 100042 | ICMV 96490-S1-15-1-2-1-3-2-P47-2 |
| 89 | ICMR 100995 | [(EERC-HS-8)-B-2-1-2-1 X ICMV 96490-S1-15-1-2-2-1-2]-28-5-2 |
| 90 | ICMR 100145 | [MRC HS-130-2-2-1-B-B-3-B-B-B-1-3-1 X ICMV 96490-S1-15-1-2-2-1-2]-12-4-3 |
| 91 | ICMR 100045 | [AIMP 92901 S1-296-2-1-1-4-2-B-15-2-1 X ICMV 96490-S1-15-1-2-3-2]-5-1-1 |
| 92 | ICMP 100030 | (AIMP 92901-S1-10-1-3XICMR 312 S1-113)-27-2-2-1-1-1 |
| 93 | ICMR 100102 | [ICMR 312 S1-3-2-1-2-2-B-B-B X ICMV 96490-S1-15-1-2-2-1-2]-12-2-1 |
| 94 | ICMR 100071 | [[(IPC 1617×SDMV 90031-S1-84-1-1-1-1)×AIMP 92901 S1-296-2-1-1-3-B-1]-4-4-2-1 X ICMV 96490-S1-15-4-1-1-2]-29-1-3 |
| 95 | ICMR 100142 | [(EERC-HS-8)-B-2-1-2-1 X ICMV 96490-S1-15-1-2-2-1-2]-29-2-2 |
| 96 | ICMR 100057 | [ICMV 96490-S1-15-1-2-1-1 X (EERC-HS-8)-B-2-1-2-1]-21-2-3 |
| 97 | ICMR 100996 | [MRC HS-130-2-2-1-B-B-3-B-B-B-1-2-1 X ICMV 96490-S1-15-1-2-1-3-1]-1-3-1 |
| 98 | ICMR 100146 | (GGP S1-79-1XAIMP 92901-S1-10-1-3)-2-2-2 |
| 99 | ICMR 100073 | [MRC HS-130-2-2-1-B-B-3-B-B-B-1-3-1 X {[(((IP 12322-1-2)×B-Lines)-B-14) × (MRC S1-156-2-1-B)]-B-1-3-3-B}×{GB 8735-S1-15-3-1-1-3-4-2-2-2-1}-B-11-5-1-1-1]-20-1-3-2-1 |
| 100 | ICMR 100152 | [MRC HS-130-2-2-1-B-B-3-B-B-B-1-3-1 X ICMV 96490-S1-15-1-2-2-1-2]-12-1-3-2-1 |
| 101 | ICMR 100154 | [[(IPC 1617×SDMV 90031-S1-84-1-1-1-1)×AIMP 92901 S1-296-2-1-1-3-B-1]-4-4-2-1 X ICMV 96490-S1-15-4-1-1-2]-29-1-2-1-B |
| 102 | ICMR 100089 | [MRC HS-130-2-2-1-B-B-3-B-B-B-1-2-1 X ICMV 96490-S1-15-1-2-1-3-1]-1-3-1-1-2 |
| 103 | ICMR 100201 | [ICMV 96490-S1-15-1-2-2-2 X LaGrap C2-S1-14-4-1-3-4-4]-24-1-2-2-1 |
| 104 | ICMR 100997 | [ICMV 96490-S1-15-4-1-1-2 X (EERC-HS-8)-B-2-1-2-1]-4-3-1-1-4 |
| 105 | ICMR 100998 | IP 9301 S1-11-3-1-3 |
| 106 | ICMR 1201 | (MC 94 C2-S1-3-2-2-2-1-3-B-B x AIMP 92901 S1-488-2-1-1-4-B-B)-B-2-2-2 |
| 107 | ICMR 1301 | ICMV 96490-S1-15-1-2-1-1 |
| 108 | ICMR 12555 | (MC 94 C2-S1-3-2-2-2-1-3-B-B x AIMP 92901 S1-488-2-1-1-4-B-B)-B-28-1-1 |
| 109 | ICMP 100410 | IP 10394 S1-4-4-1-1-1 |
| 110 | ICMR 100109 | IP 9301 S1-11-3-1-3-1 |
| 111 | ICMR 100216 | IP 17707 S1-5-4-2-2-1 |
| 112 | ICMP 100411 | IP 17620 S1-3-3-4-2-1 |
| 113 | ICMP 100412 | IP 17690 S1-10-1-1-1-1 |
| 114 | ICMP 100413 | IP 11584 S1-4-1-1-2-1 |
| 115 | ICMP 100434 | IP No. 9268-13-4-4-B-B-B |
| 116 | ICMP 100435 | IP No. 17635-12-1-4-B-2-2 |
| 117 | ICMP 100436 | IP No. 9438-2-2-5-B-B-B |
| 118 | ICMP 100437 | IP No. 9438-11-2-5-B-1-1 |
| 119 | ICMP 100438 | IP No. 17701-2-6-5-B-2-B |
| 120 | ICMP 100439 | IP No. 17624-3-2-2-B-4-B |
| 121 | ICMP 100440 | IP No. 17624-5-6-1-B-1-B |
| 122 | ICMP 100430 | [(EEBC S1-407-1-B-B-B-B-B-1-B-1-B-5-1x 3981-3989 G1)-2-1-1x(ICMB 04888 x ICMB 02333)-3-1-3-1]-13-3-2-1-1 |
| 123 | ICMR 100931 | (EERC-HS-23)-B-3-1-2-4-B |
| 124 | ICMP 100431 | ICMR 312 S1-8-1-1-1-1-B-B-B-1-B-22-3x R-lines bulk (20216-20249/K09)]-1 |
| 125 | ICMP 100432 | [AIMP 92901 S1-296-2-1-1-4-2-B-15-2-1 X ICMV 96490-S1-15-1-2-3-2]-30-1-1-3-1 |
| 126 | ICMP 100433 | IP No. 9438-11-2-2-B-1-B |
| 127 | ICMP 100415 | (ICTP 8203 S1-361-B-B-10-1XAIMP 92901-S1-278-2-6)-8-1-2-1 |
| 128 | ICMP 100416 | (ICTP 8203 S1-361-B-B-10-1XAIMP 92901-S1-278-2-6)-8-4-3-1 |
| 129 | ICMP 100417 | (ICMR 312 S1-61-2-3XICTP 8203 S1-361-B-B-1-1)-18-1-2-2 |
| 130 | ICMP 100418 | (AIMP 92901-S1-10-1-3XICMR 312 S1-113)-5-1-2-1 |
| 131 | ICMP 100419 | (AIMP 92901-S1-10-1-3XICMR 312 S1-113)-7-2-4-2 |
| 132 | ICMP 100420 | (ICMV 221 S1 - 84XICMR 312 S1-59-2-3)-11-1-1-1 |
| 133 | ICMP 100421 | [EEDBC S1-2-1-1-1-1-1-1-B-B-2-1-B X ICMR 312 S1-113]-37-2-1-1 |
| 134 | ICMP 100422 | [(EEBC S1-407-1-B-B-B-B-B-1-B-1-B-5-1 x B-bulk (3981-3989/S06 G1))-2-1-3 X AIMP 92901-S1-278-1-1]-16-6-3-1 |
| 135 | ICMP 100423 | [(AIMP 92901 S1-480-1-1-1-2-B-2 x ICMR 312 S1-3-2-3-2-1-1-B-B)-B-9-2-3 X CGP S1-67-1]-8-6-1-3 |
| 136 | ICMP 100424 | (ICMR 312 S1-136XGGP S1-38-2)-17-3-1-2-1 |
| 137 | ICMP 100425 | (ICMR 312 S1-136XGGP S1-38-2)-17-3-2-2-1 |
| 138 | ICMP 100426 | (AIMP 92901-S1-278-1-4XICMR 312 S1-59-2-3)-32-2-2-2-2 |
| 139 | ICMR 100208 | (GGP S1-79-1XAIMP 92901-S1-10-1-3)-4-4-1-1-1 |
| 140 | ICMR 100236 | (ICMR 312 S1-61-2-3XICTP 8203 S1-361-B-B-1-1)-18-3-1-1-3 |
| 141 | ICMP 100427 | (ICTP 8203 S1-361-B-B-10-1XICMR 312 S1-59-1-1)-16-4-1-3-1 |
| 142 | ICMP 100429 | (IP No. 8950-1XAIMP 92901-S1-10-1-3)-39-2-1-1-3 |
| 143 | ICMB 100077 | (ICMB 94333 x ICMB 01222)-47-1-B-B-B |
| 144 | ICMB 100633 | (ICMB 04888 x ICMB 02444)-1-4-B-1-2-2-2-2 |
| 145 | ICMB 100635 | ({(MC 94 S1-34-1-B x HHVBC)-16-2-1-1-1-1-B-B-5 x (MC 94 S1-34-1-B x HHVBC)-10-4-1-2-1-B-B-1-30-2-4-3-6-4-3 x (ICMB 04888 x ICMB 02333)-3-1-2-2)-34-5-1-1 |
| 146 | ICMB 100636 | ({(MC 94 S1-34-1-B x HHVBC)-16-2-1-1-1-1-B-B-5 x (MC 94 S1-34-1-B x HHVBC)-10-4-1-2-1-B-B-1-30-2-4-3-6-4-3 x (ICMB 04888 x ICMB 02333)-3-1-2-2)-34-6-1-1 |
| 147 | ICMB 100637 | (ICMB 98222 x (EEBC S1-407-1-B-B-B-B-B-1-B-1-B-13-1 x B-bulk (3981-3989/S06 G1))-1-2-3)-22-4-2-1 |
| 148 | ICMB 100638 | [{(81B x 4017-6-1-1)-3-1-4-3-4-1-2-1-B-2-2 x ICMB 05888}x (HHVDBC Medium HS-120-1-2-1-1-1-1 x HHVDBC Medium HS-15-1-1-1-2-2-4)]-19-2-2-4-1-B |
| 149 | ICMB 100198 | [{(81B x 4017-6-1-1)-3-1-4-3-4-1-2-1-B-2-2 x ICMB 05888}x (HHVDBC Medium HS-120-1-2-1-1-1-1 x HHVDBC Medium HS-15-1-1-1-2-2-4)]-19-2-4-1-B |
| 150 | ICMB 100639 | ((SRC II C3 S1-19-3-2 x HHVBC)-27-1-3-3-3-3-2 x {[(843B x ICTP 8202-161-5)-20-3-B-B-3 x B-bulk]-2-B-1-2-2-B-B-B-11-1 x B-bulk (3981-4011/S06 G1)}-3-2-4-4)-35-2-4-1 |
| 151 | ICMB 100641 | [(EEBC S1-407-1-B-B-B-B-B-1-B-1-B-5-1x 3981-3989 G1)-2-1-1x(ICMB 04888 x ICMB 02333)-3-1-3-1]-20-1-2-3-1-1 |
| 152 | ICMB 100642 | [(EEBC S1-407-1-B-B-B-B-B-1-B-1-B-5-1x 3981-3989 G1)-2-1-1x{[(843B x ICTP 8202-161-5)-20-3-B-B-3 x B-bulk]-2-B-9 x [(ICMB 96555 x LaGrap C2 S1-32-1)-10 x IP 14758-2-1]-8-2}-1-1-2-2]-2-1-2-2-1-1 |
| 153 | ICMB 100643 | [(EEBC S1-407-1-B-B-B-B-B-1-B-1-B-13-1 x B-bulk (3981-3989/S06 G1))-1-2-3x[(ICMR 312 S1-1-5-2-B x HHVBC)-10-2-1-2-3 x EEBC 407)-7-2-1-3]-14-3-2-1-B-1 |
| 154 | ICMB 100644 | [(ICMB 95111 x EEBC S1-407-1-B-B)-17-3-1-B-B-B-B-4-Bx 3981-4011 G2}-1-4-2x[(ICMR 312 S1-1-5-2-B x HHVBC)-10-2-1-2-3 x EEBC 407)-7-2-1-3]-9-5-1-5-2-2 |
| 155 | ICMB 100645 | [(ICMB 95111 x EEBC S1-407-1-B-B)-17-3-1-B-B-B-B-4-Bx 3981-4011 G2}-1-4-2x{EEBC S1-407-1-B-B-B-B-B-1-B-1-B-5-1x 3981-3989 G1}-2-1-3]-16-3-1-1-1-1 |
| 156 | ICMB 100617 | (ICMB 98222 x (EEBC S1-407-1-B-B-B-B-B-1-B-1-B-13-1 x B-bulk (3981-3989/S06 G1))-1-2-3)-40-3-1 |
| 157 | ICMB 100618 | ({(MC 94 S1-34-1-B x HHVBC)-16-2-1-1-1-1-B-B-5 x (MC 94 S1-34-1-B x HHVBC)-10-4-1-2-1-B-B-1-30-2-4-2-1-3-4 x (ICMB 04888 x ICMB 02333)-3-1-2-2)-55-2-3 |
| 158 | ICMB 100619 | ({(MC 94 S1-34-1-B x HHVBC)-16-2-1-1-1-1-B-B-5 x (MC 94 S1-34-1-B x HHVBC)-10-4-1-2-1-B-B-1-30-2-4-2-6-3-2 x {[(843B x ICTP 8202-161-5)-20-3-B-B-3 x B-bulk]-2-B-9 x [(ICMB 96555 x LaGrap C2 S1-32-1)-10 x IP 14758-2-1]-8-2}-1-1-1-2)-44-1-1 |
| 159 | ICMB 100620 | (ICMB 98222 x {EEBC S1-407-1-B-B-B-B-B-1-B-1-B-10-1x 3981-3989 G1}-2-4-1)-31-7-2 |
| 160 | ICMB 100621 | (ICMB 98222 x {EEBC S1-407-1-B-B-B-B-B-1-B-1-B-10-1x 3981-3989 G1}-2-4-1)-32-8-3 |
| 161 | ICMB 100622 | ([78-7088/3/SER3 AD//B282/(3/4)EB x PBLN/S95-359]-7-4-B-B-2-B-BxMRC HS-130-2-2-1-B-B-3-B-B-B-1-3-1)-46-3-1 |
| 162 | ICMB 100623 | (ICMB 98222xMRC HS-130-2-2-1-B-B-3-B-B-B-1-3-1)-6-2-1 |
| 163 | ICMB 100624 | ({(MC 94 S1-34-1-B x HHVBC)-16-2-1-1-1-1-B-B-5 x (MC 94 S1-34-1-B x HHVBC)-10-4-1-2-1-B-B-1-30-2-4-3-6-4-3 x (ICMB 04888 x ICMB 02333)-3-1-2-2)-17-9-1 |
| 164 | ICMB 100625 | ((ICMB 99555 x ICMB 99111)-2-1-1-B-B-B-5 x (NC D2 BC7F4-34-3-1-2-B-2-B x EEBC 407)-4-2-2-2)-15-2-1 |
| 165 | ICMB 100626 | ({(MC 94 S1-34-1-B x HHVBC)-16-2-1-1-1-1-B-B-5 x (MC 94 S1-34-1-B x HHVBC)-10-4-1-2-1-B-B-1-30-2-4-2-6-3-2 x {[(843B x ICTP 8202-161-5)-20-3-B-B-3 x B-bulk]-2-B-9 x [(ICMB 96555 x LaGrap C2 S1-32-1)-10 x IP 14758-2-1]-8-2}-1-1-1-2)-46-3-1 |
| 166 | ICMB 100627 | (ICMB 98222 x {EEBC S1-407-1-B-B-B-B-B-1-B-1-B-10-1x 3981-3989 G1}-2-4-1)-29-3-1 |
| 167 | ICMB 100628 | ({(MC 94 S1-34-1-B x HHVBC)-16-2-1-1-1-1-B-B-5 x (MC 94 S1-34-1-B x HHVBC)-10-4-1-2-1-B-B-1-30-2-4-2-1-3-4 x (ICMB 04888 x ICMB 02333)-3-1-2-2)-22-3-1 |
| 168 | ICMB 100629 | ({(MC 94 S1-34-1-B x HHVBC)-16-2-1-1-1-1-B-B-5 x (MC 94 S1-34-1-B x HHVBC)-10-4-1-2-1-B-B-1-30-2-4-2-1-3-4 x (ICMB 04888 x ICMB 02333)-3-1-2-2)-37-5-2 |
| 169 | ICMB 100630 | ({(MC 94 S1-34-1-B x HHVBC)-16-2-1-1-1-1-B-B-5 x (MC 94 S1-34-1-B x HHVBC)-10-4-1-2-1-B-B-1-30-2-4-2-6 x {[(843B x ICTP 8202-161-5)-20-3-B-B-3 x B-bulk]-2-B-9 x [(ICMB 96555 x LaGrap C2 S1-32-1)-10 x IP 14758-2-1]-8-2}-1-1-1-2)-38-2-3 |
| 170 | ICMB 100631 | (EEDBC S1-425-2-1-2-3-B-1-B-3-1x 3981-4011 G2}-2-4-2 x (EEBC S1-407-1-B-B-B-B-B-1-B-1-B-13-1 x B-bulk (3981-3989/S06 G1))-1-2-3)-24-1-2 |
| 171 | ICMR 100999 | [MRC HS-130-2-2-1-B-B-3-B-B-B-1-3-1 X ICMV 96490-S1-15-1-2-2-1-2]-61-6-1-1 |
| 172 | ICMR 101000 | [MRC HS-130-2-2-1-B-B-3-B-B-B-1-3-1 X ICMV 96490-S1-15-1-2-2-1-2]-86-7-3-1 |
| 173 | ICMR 101001 | [ICMV 96490-S1-15-1-4-3-1 X MRC HS-130-2-2-1-B-B-3-B-B-B-1-3-1]-68-1-1-1 |
| 174 | ICMR 100544 | [MRC HS-130-2-2-1-B-B-3-B-B-B-1-3-1 X {[(((IP 12322-1-2)×B-Lines)-B-14) × (MRC S1-156-2-1-B)]-B-1-3-3-B}×{GB 8735-S1-15-3-1-1-3-4-2-2-2-1}-B-11-5-1-1-1]-20-1-3-1-2-1 |
| 175 | ICMR 100568 | [MRC HS-130-2-2-1-B-B-3-B-B-B-1-3-1 X {[(((IP 12322-1-2)×B-Lines)-B-14) × (MRC S1-156-2-1-B)]-B-1-3-3-B}×{GB 8735-S1-15-3-1-1-3-4-2-2-2-1}-B-11-5-1-1-1]-20-1-3-1-3-1-2 |
| 176 | ICMR 101003 | [MRC HS-130-2-2-1-B-B-3-B-B-B-1-3-1 X ICMV 96490-S1-15-1-2-2-1-2]-11-2-2-2-1-1 |
| 177 | ICMR 101004 | [MRC HS-130-2-2-1-B-B-3-B-B-B-1-3-1 X ICMV 96490-S1-15-1-2-2-1-2]-11-3-1-2-4-2 |
| 178 | ICMR 101005 | [ICMV 96490-S1-15-1-2-2-2 X ICMV 96490-S1-15-4-1-1-2]-10-2-1-1-2-1 |
| 179 | ICMR 101006 | [ICMV 96490-S1-15-1-2-2-2 X LaGrap C2-S1-14-4-1-3-4-4]-6-1-1-2-1-2 |
| 180 | ICMR 100550 | [[(IPC 1617×SDMV 90031-S1-84-1-1-1-1)×AIMP 92901 S1-296-2-1-1-3-B-1]-4-4-2-1 X ICMV 96490-S1-15-4-1-1-2]-3-2-1-3-5-1 |
| 181 | ICMR 101007 | [MRC HS-130-2-2-1-B-B-3-B-B-B-1-2-1 X {[(((IP 12322-1-2)×B-Lines)-B-14) × (MRC S1-156-2-1-B)]-B-1-3-3-B}×{GB 8735-S1-15-3-1-1-3-4-2-2-2-1}-B-11-5-1-1-1]-17-2-1-4-1-1 |
| 182 | ICMR 101008 | [ICMV 96490-S1-15-1-4-3-1 X HHVBC Tall S1-51-1-P1-3-B]-17-2-1-2-1-2 |
| 183 | ICMR 100640 | [ICMV 96490-S1-15-1-4-3-1 X HHVBC Tall S1-51-1-P1-3-B]-17-2-1-2-2-1-4 |
| 184 | ICMR 101010 | [(EERC-HS-8)-B-2-1-2-1 X ICMV 96490-S1-15-1-2-2-1-2]-30-1-2-3-2-3 |
| 185 | ICMR 101011 | [LaGrap C2-S1-14-4-1-3-4-1 X (MC 94 C2-S1-3-2-2-2-1-3-B-B x SDMV 90031 S1-93-3-1-1-3-2-B-2)-B-23-2-1]-8-6-1-2-3-1 |
| 186 | ICMR 101012 | [ICMV 96490-S1-15-1-2-1-1 X (EERC-HS-8)-B-2-1-2-1]-2-3-2-2-1-1 |
| 187 | ICMR 101013 | [ICMV 96490-S1-15-1-2-1-1 X (EERC-HS-8)-B-2-1-2-1]-21-2-2-2-2-3 |
| 188 | ICMR 100556 | [ICMV 96490-S1-15-4-1-1-2 X (EERC-HS-8)-B-2-1-2-1]-4-3-1-1-4-1 |
| 189 | ICMR 101014 | [AIMP 92901 S1-296-2-1-1-4-2-B-15-2-1 X ICMV 96490-S1-15-1-2-3-2]-5-1-2-1-1-1 |
| 190 | ICMR 101015 | [AIMP 92901 S1-296-2-1-1-4-2-B-15-2-1 X ICMV 96490-S1-15-1-2-3-2]-6-3-3-3-3-1 |
| 191 | ICMR 101016 | [MRC HS-130-2-2-1-B-B-3-B-B-B-1-2-1 X ICMV 96490-S1-15-1-2-1-3-1]-1-3-1-1-4-2 |
| 192 | ICMR 101017 | [(EERC-HS-8)-B-2-1-2-1 X ICMV 96490-S1-15-1-2-1-3-1]-2-3-1-2-2-2 |
| 193 | ICMB 100652 | (HTBLN/95-98 x ICMB 89111)-3-B-B-3-B-B-B-B-B-3 |
| 194 | ICMB 100653 | (ICMB 04888-3x ICMB 98222)-10-2-2-1-1 |
| 195 | ICMB 100671 | (ICMB 04888 x HHVDBC HS-10-1-2-1-1-1-2-B)-12-3-1-1 |
| 196 | ICMB 100463 | (ICMB 99555 x ICMB 00555)-5-4-3-B-B-4-4 |
| 197 | ICMB 100244 | (EEDBC S1-425-2-1-2-3-B-1-B-3-1x 3981-4011 G2}-2-4-2 x (EEBC S1-407-1-B-B-B-B-B-1-B-1-B-13-1 x B-bulk (3981-3989/S06 G1))-1-2-3)-9-3 |
| 198 | ICMB 100302 | (EEDBC S1-425-2-1-2-3-B-1-B-3-1x 3981-4011 G2}-2-4-2 x (EEBC S1-407-1-B-B-B-B-B-1-B-1-B-13-1 x B-bulk (3981-3989/S06 G1))-1-2-3)-9-4-1 |
| 199 | ICMR 100041 | (MC 94 C2-S1-3-2-2-2-1-3-B-B x ICMR 312 S1-3-2-3-2-1-1-B-B)-B-23-3-1-2-1x R-lines bulk (20216-20249/K09)] |
| 200 | ICMR 100990 | [MC 94 C2-S1-3-1-3-1-2-2-2-B-B-1 x (ICMV-IS 94206-7 × (SRC II C3 S1-1-1-2 x HHVBC)-1-3-3))-B-10-1-1-3-3-2]-B-13-2-1 |
| 201 | ICMR 1203 | AIMP 92901 S1-296-2-1-1-1-B-B-6-B-B |
| 202 | ICMR 1501 | [(IPC 1617×SDMV 90031-S1-84-1-1-1-1)×AIMP 92901 S1-296-2-1-1-3-B-1]-4-4-5-3-2 |
| 203 | ICMR 1502 | MRC HS-130-2-2-1-B-B-3-B-B-B-1-3-1 |
| 204 | ICMR 1503 | (MC 94 C2-S1-3-2-2-2-1-3-B-B x SDMV 90031 S1-93-3-1-1-3-2-B-2)-B-23-2-1 |
| 205 | ICMB 1504 | {[(BESCBPT/91-40 x SPF3/S91-3)-1-2-2-3 x B-bulk]-8-1-1-3-B-B-B-B-3-1 x B-bulk (3981-4011/S06 G1)}-1-3-2 |
| 206 | ICMR 1505 | JBV 3 S1 -237-1-3-3-1-B |
| 207 | ICMR 1202 | (MC 94 C2-S1-3-2-2-2-1-3-B-B x ICMR 312 S1-3-2-3-2-1-1-B-B)-B-34-4-1 |
| 208 | ICMB 100195 | (MC 94 C2-S1-3-2-2-2-1-3-B-B x ICMR 312 S1-3-2-3-2-1-1-B-B)-B-34-4-1-d2-3-2-1 |
| 209 | IPC 21 | WC 2-8-1 |
| 210 | IPC 492 | (B 282 x J 804-1-3-9)-7-2-2 |
| 211 | IPC 337 | (5054B x F4FC 1498-1-1-2)-7-1-1-1 |
| 212 | IPC 616 | (J 260-1 x 700557-1-4-10-5-1)-1-2-1-3 |
| 213 | IPC 689 | R-294-1-2-8-2 |
| 214 | IPC 716 | (LCSN 282-4-1-1 x S10B-38)-15-2-1 |
| 215 | IPC 1027 | (J 25-1 x J 1798)-1-1-24-2-1-3 |
| 216 | IPC 736 | {(SC14(M) x [(SD2 x EB 2) x (D 1088-1)]}-64 |
| 217 | IPC 774 | [{(J934x700544+)x(J1644x700490+)}x{G75-FS+x(J1623x700544+)}]-4-1-5-2 |
| 218 | IPC 417 | {G73-FS-41 x (J 1188 x Cassady)}-5-6-1-2 |
| 219 | IPC 835 | (F4FC 1436-4-3-2 x J 104 ST)-22-1-1 |
| 220 | IPC 1047 | [(J 1623 x 700490-2-6) x (EC 298-2-5-23)]-2-4 |
| 221 | IPC 954 | (E 298 x F4FC 1498-1-1-2)-5-3-3-1 |
| 222 | IPC 957 | [(700626-21) x (B 282-2-1 x 700651-1)]-4-7-1 |
| 223 | IPC 367 | (E 298 x F4FC 1498-1-1-2)-6-3 |
| 224 | IPC 1025 | (J 1248 x 700112)-1-2-31-1-1-2 |
| 225 | IPC 1040 | (700619 x 700599)-3-1-3-6-1-5 |
| 226 | IPC 1189 | [(L 108-1) x (J 937 x 700797-16-2-2)]-5-1-1 |
| 227 | IPC 1329 | (NEP 7-5603 x SS 48-40)-4-6 |
| 228 | IPC 1444 | (B Senegal-2-5 x 700651)-2-1-1 |
| 229 | IPC 1470 | (B 282 x J 104)-12-B-B-B-B |
| 230 | IPC 1485 | (842B x 3/4EB-100)-11-9-2-50-B-B-1 |
| 231 | IPC 1466 | H 77/833-2 |
| 232 | IPC 1503 | {K 560-2 x (J 934-7 x 700544-7-2-1)}-4-1-3-3-2-2 |
| 233 | IPC 1536 | {(B 282 x S10B-38)-30-2-2-2 x Togo-29-2-2}-32-2 |
| 234 | IPC 795 | (LCSN 72-1-2-2 x S10B-106)-2-2-1 |
| 235 | ICMB 92111 | (81B x 843B)-11-1-1-B |
| 236 | ICMB 92888 | (843B x ICMPS 900-9-3-2-2)-41-2-6-2-2 |
| 237 | ICMB 96666 | (SPF3/S91-327 x SPF3/S91-5)-6-2-3 |
| 238 | ICMB 97111 | HTBC HS-48-B-1-1-1-1 |
| 239 | ICMB 98666 | (ICMB 89111 x IPC 1466)-21-1-3-6-B-5 |
| 240 | ICMB 01444 | [{843B x (B 816 x 3/4 EB-105-6-1)-3-3}-15-B-4-1]-12-2-4-1 |
| 241 | ICMB 01888 | {[(81B x SRL-53-1) x 843B]-3-5-3 x [(843B x 111B)-10-1-2-2]}-226-B-2-B-B-B |
| 242 | ICMB 02555 | ICMV 87901-175-2-3-2-B-1 |
| 243 | ICMB 03555 | (843B x ICTP 8202-161-5)-17-1-3-B-2 |
| 244 | ICMB 03666 | DMR1 S2-52-4-1-1-B |
| 245 | ICMB 04555 | [D2BLN/95-214 x (ICMB 96333 x HHVBC)]-11-B-2 |
| 246 | ICMB 05666 | [(BSECBPT/91-40 x SPF3/S91-3)-1-2-2-3 x B-bulk]-2-B-1-1 |
| 247 | ICMB 06555 | [{{{843B x (843B x 700651)-11-1-2-B} x 1163B} x ICMB 89111x ICMB 88005)}-27+ x B-bulk]-3-B-B-10 |
| 248 | ICMB 06888 | [(BSECBPT/91-40 x SPF3/S91-514)-7-2-1-B x B-bulk]-2-B-1-1 |
| 249 | ICMB 07333 | [ICMB 97444 x (D2BLN/95-98 x EEBC C1-1)-7-B-B]-34-2-4-B-B |
| 250 | ICMB 07777 | {ICMB 99555 x [(78-7088/3/SER3 AD//B282/(3/4 EB) x PBLN/S95-359)-19-5-B-B]}-13-2-B-B-B-B |
| 251 | ICMB 07999 | (HTBC 48-B-1-1-1-5 x B-bulk)-25-1-B-B |
| 252 | ICMB 08555 | (ICMB 96555 x IP 10437)-2-4-2-B-6-1 |
| 253 | ICMB 10888 | [ICMB 99555 x {78-7088/3/SER3 AD//B282/(3/4)EB x PBLN/S95-359}-10-2-B-2]-18-3-B-B-B-B |
| 254 | ICMB 11555 | [(MC 94 S1-81-1-B x HHVBC)-4-4-1 x (MC 94 S1-81-1-B x HHVBC)-4-2-4]-10-3-1-B-1 |
| 255 | ICMB 11777 | [ICMB 97444 x (843B x 405B)-4]-1 -2-B-B-B-B |
| 256 | ICMB 11999 | (DMR 133 x HTBC 48-B-1-1-1-5)-9-1-B-B-1 |
| 257 | ICMB 14111 | (HTBLN/95-98 x ICMB 89111)-8-B-B-B-B-2-B |
| 258 | ICMB 14444 | (B x B) F2 (G-6)-86-1-1-3 |
| 259 | ICMB 15777 | IC-CZBC-C0-26-3-1-1-B-2 |
| 260 | ICMR 06999 | MRC S1-4-1-3-B-B-B-B |
| 261 | ICMR 07666 | ICMS 7704-S1-126-5-2-1-3-2-2-2-B-3 |
| 262 | ICMR 07777 | Jakhrana × ESRC II S2-81-3-2-2-2 |
| 263 | ICMR 07888 | (RCB-2-S1-138-1-3 × MRC)-B-2-1-2-B |
| 264 | ICMR 08111 | (ICMS 7704-S1-127-5-1 × RCB-2 Tall )-B-19-3-4-5-3 |
| 265 | ICMR 08333 | RCB-2 S1-19-2-2-1-2-3-2-1-B-B-B |
| 266 | IPC 186 | (J 260-1 x 700557-1-4-10-5-1-1)-2-2-1 |
| 267 | ICMR 08888 | ICMS 7704-S1-52-3-1-2-1-2-1-6-B-B |
| 268 | ICMR 08999 | JBV 3 S1-18-2-2-1-3-2 |
| 269 | ICMR 09333 | MRC HS-225-3-5-2-B-B-B-B-B |
| 270 | ICMB 07888 | [HTBLN/95-98 x (SPF3/S91-544 x SPF3/S91-5)-5-1-2]-3-B-B-1-B-1-B |
| 271 | ICMR 09666 | [(((IP 12322-1-2)×B-Lines)-B-14) × (MRC S1-156-2-1-B)]-B-1-3-3-B-B |
| 272 | ICMR 10222 | ((ICMV IS 94206 S1-15-2)×{(SRC II C3 S1-19-3-2 x HHVBC)-5-3-1})-B-13-4-2-1-1-1-1-3-2 |
| 273 | ICMR 10888 | ICMV 93074 S1-9-1-1-1-3-B-B-B-B |
| 274 | ICMR 11666 | ICMV 91059 S1-4-2-3-2-1-1-4-B-1-3-B-1 |
| 275 | ICMR 12333 | (E 298 x LCSN 282-4-1-2)-12-2-1-2-B-B-B-1 |
| 276 | ICMR 12444 | (ICMS 7704-S1-127-5-1 × RCB-2 Tall )-B-19-3-2-1-1-1-B |
| 277 | ICMR 12777 | [(IPC 1617×SDMV 90031-S1-84-1-1-1-1)×GB 8735-S1-25-4-4-1-1-3-1-1]-1-1-3-2-1-B-B |
| 278 | ICMR 16111 | (ICMS 7704-S1-18-2-2 × RCB Short)-B-7-2-2-3-4-4-1 |
| 279 | ICMR 16555 | {(SRC II C3 S1-19-3-2 X HHVBC)-1-5-1} X {[((96111b X 4017-6-1-1)-1-4-4-3) X (IP 19626-4-1-2-1)]-B-6}-B-5-1-1-3 |
| 280 | ICMR 16888 | (IPC 1268 ×ICMV 91059 S1-58-2-2-2-1)-8-1-1 |
| 281 | ICMR 16999 | (IPC 107 ×SDMV 90031-S1-84-1-1-1-1)-1-2-1-2-2-B-B |

**
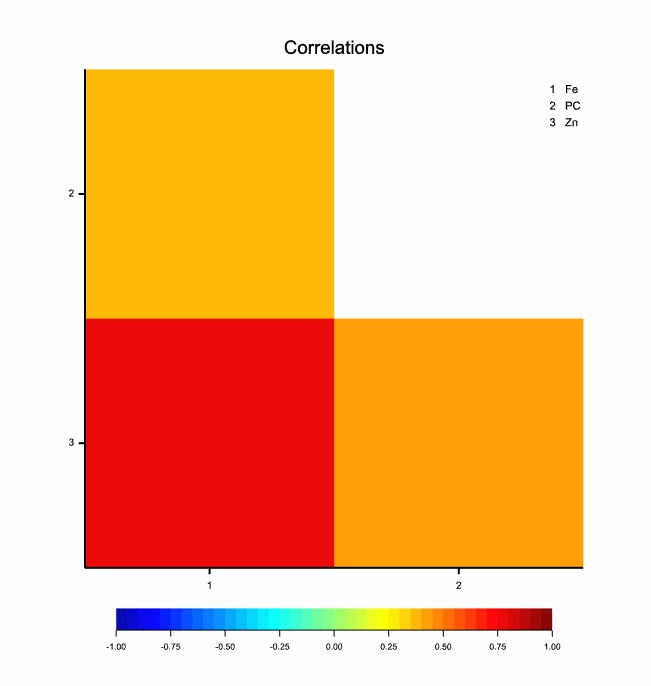
**

Figure S1. Correlation among iron (Fe), zinc (Zn) and Protein content (PC) in grains.

**
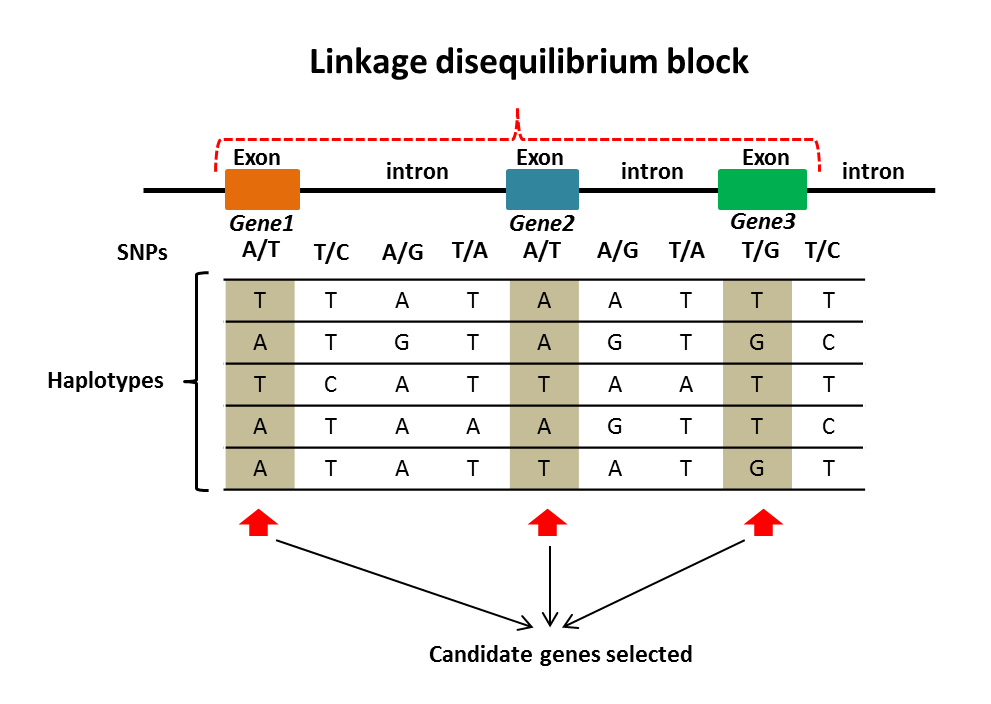
**

Figure S2: Linkage disequilibrium block of pearl millet

**Table S5. Mean performances of all the 281 inbred lines evaluated for iron (Fe), zinc (Zn) and protein content (PC)**

| **TRT No** | **Designation** | **Fe**  (mg kg^-1^) | **Zn**  (mg kg^-1^) | **Protein**  (%) |
| --- | --- | --- | --- | --- |
| 1 | ICMB 100646 | 83 | 44 | 10 |
| 2 | ICMB 100485 | 81 | 38 | 13 |
| 3 | ICMB 100648 | 120 | 71 | 13 |
| 4 | ICMB 100649 | 99 | 54 | 12 |
| 5 | ICMB 100650 | 94 | 49 | 12 |
| 6 | ICMB 100651 | 48 | 38 | 10 |
| 7 | ICMB 100219 | 80 | 51 | 11 |
| 8 | ICMB 100654 | 83 | 49 | 11 |
| 9 | ICMB 100454 | 109 | 53 | 14 |
| 10 | ICMB 100409 | 44 | 32 | 10 |
| 11 | ICMB 100655 | 65 | 46 | 10 |
| 12 | ICMP 100409 | 51 | 55 | 10 |
| 13 | ICMB 100656 | 91 | 47 | 11 |
| 14 | ICMB 100657 | 73 | 40 | 10 |
| 15 | ICMB 100465 | 95 | 50 | 11 |
| 16 | ICMB 100658 | 91 | 45 | 10 |
| 17 | ICMB 100025 | 74 | 46 | 11 |
| 18 | ICMB 100659 | 96 | 48 | 11 |
| 19 | ICMB 100660 | 74 | 45 | 12 |
| 20 | ICMB 100480 | 72 | 48 | 11 |
| 21 | ICMB 100661 | 82 | 45 | 11 |
| 22 | ICMB 100075 | 78 | 45 | 12 |
| 23 | ICMB 100662 | 61 | 50 | 11 |
| 24 | ICMB 100663 | 99 | 54 | 14 |
| 25 | ICMB 100664 | 106 | 58 | 13 |
| 26 | ICMB 100665 | 69 | 52 | 13 |
| 27 | ICMB 100666 | 84 | 45 | 11 |
| 28 | ICMB 100667 | 104 | 57 | 12 |
| 29 | ICMB 100668 | 102 | 53 | 12 |
| 30 | ICMB 100669 | 80 | 43 | 11 |
| 31 | ICMB 1503 | 72 | 42 | 11 |
| 32 | ICMB 100455 | 95 | 46 | 12 |
| 33 | ICMB 100081 | 68 | 45 | 10 |
| 34 | ICMB 100670 | 86 | 49 | 14 |
| 35 | ICMB 100672 | 74 | 41 | 13 |
| 36 | ICMB 100673 | 82 | 51 | 13 |
| 37 | ICMB 100411 | 73 | 45 | 11 |
| 38 | ICMB 100235 | 87 | 46 | 11 |
| 39 | ICMB 100304 | 78 | 38 | 10 |
| 40 | ICMB 100305 | 83 | 50 | 11 |
| 41 | ICMB 100674 | 52 | 42 | 10 |
| 42 | ICMB 100675 | 90 | 58 | 11 |
| 43 | ICMB 100677 | 86 | 49 | 13 |
| 44 | ICMB 100678 | 79 | 40 | 10 |
| 45 | ICMB 100679 | 86 | 50 | 10 |
| 46 | ICMB 100680 | 113 | 65 | 13 |
| 47 | ICMB 100248 | 78 | 42 | 9 |
| 48 | ICMB 100252 | 78 | 51 | 10 |
| 49 | ICMP 100445 | 77 | 51 | 14 |
| 50 | ICMB 02333 | 81 | 43 | 12 |
| 51 | ICMB 99222 | 87 | 45 | 13 |
| 52 | ICMR 100973 | 82 | 49 | 12 |
| 53 | ICMR 100974 | 66 | 45 | 14 |
| 54 | ICMR 13777 | 74 | 61 | 11 |
| 55 | ICMR 100327 | 63 | 39 | 9 |
| 56 | ICMR 100300 | 88 | 47 | 12 |
| 57 | ICMR 06555 | 91 | 48 | 13 |
| 58 | ICMR 100725 | 106 | 59 | 14 |
| 59 | ICMR 100975 | 61 | 42 | 10 |
| 60 | ICMR 100444 | 74 | 44 | 11 |
| 61 | ICMR 100976 | 80 | 41 | 11 |
| 62 | ICMR 100977 | 68 | 47 | 11 |
| 63 | ICMR 100978 | 115 | 87 | 14 |
| 64 | ICMR 100979 | 65 | 32 | 14 |
| 65 | ICMR 100920 | 54 | 37 | 11 |
| 66 | ICMR 100299 | 92 | 56 | 12 |
| 67 | ICMP 100239 | 68 | 57 | 12 |
| 68 | ICMR 100981 | 94 | 66 | 12 |
| 69 | ICMR 100982 | 71 | 35 | 11 |
| 70 | ICMR 100983 | 60 | 39 | 10 |
| 71 | ICMR 100984 | 76 | 40 | 12 |
| 72 | ICMR 100985 | 81 | 46 | 10 |
| 73 | ICMR 100445 | 75 | 47 | 13 |
| 74 | ICMR 100986 | 86 | 51 | 12 |
| 75 | ICMR 100987 | 78 | 43 | 11 |
| 76 | ICMR 100988 | 84 | 49 | 13 |
| 77 | ICMR 100019 | 72 | 48 | 13 |
| 78 | ICMR 100989 | 71 | 45 | 13 |
| 79 | ICMR 100991 | 84 | 45 | 12 |
| 80 | ICMR 100992 | 96 | 41 | 12 |
| 81 | ICMR 100028 | 89 | 57 | 14 |
| 82 | ICMR 100993 | 64 | 39 | 9 |
| 83 | ICMR 100994 | 70 | 43 | 10 |
| 84 | ICMR 100056 | 87 | 54 | 13 |
| 85 | ICMR 100038 | 89 | 48 | 12 |
| 86 | ICMR 100040 | 96 | 48 | 12 |
| 87 | ICMR 100098 | 74 | 54 | 9 |
| 88 | ICMR 100042 | 92 | 44 | 11 |
| 89 | ICMR 100995 | 87 | 50 | 12 |
| 90 | ICMR 100145 | 90 | 47 | 11 |
| 91 | ICMR 100045 | 76 | 42 | 11 |
| 92 | ICMP 100030 | 83 | 57 | 11 |
| 93 | ICMR 100102 | 114 | 56 | 13 |
| 94 | ICMR 100071 | 104 | 57 | 11 |
| 95 | ICMR 100142 | 105 | 48 | 13 |
| 96 | ICMR 100057 | 87 | 47 | 12 |
| 97 | ICMR 100996 | 84 | 44 | 10 |
| 98 | ICMR 100146 | 93 | 60 | 13 |
| 99 | ICMR 100073 | 96 | 56 | 11 |
| 100 | ICMR 100152 | 110 | 59 | 12 |
| 101 | ICMR 100154 | 87 | 45 | 12 |
| 102 | ICMR 100089 | 86 | 45 | 11 |
| 103 | ICMR 100201 | 84 | 44 | 12 |
| 104 | ICMR 100997 | 86 | 43 | 11 |
| 105 | ICMR 100998 | 100 | 54 | 12 |
| 106 | ICMR 1201 | 68 | 35 | 10 |
| 107 | ICMR 1301 | 88 | 43 | 12 |
| 108 | ICMR 12555 | 79 | 48 | 11 |
| 109 | ICMP 100410 | 113 | 54 | 12 |
| 110 | ICMR 100109 | 100 | 50 | 10 |
| 111 | ICMR 100216 | 86 | 50 | 13 |
| 112 | ICMP 100411 | 80 | 55 | 12 |
| 113 | ICMP 100412 | 83 | 67 | 12 |
| 114 | ICMP 100413 | 79 | 39 | 12 |
| 115 | ICMP 100434 | 101 | 68 | 14 |
| 116 | ICMP 100435 | 94 | 52 | 13 |
| 117 | ICMP 100436 | 112 | 63 | 15 |
| 118 | ICMP 100437 | 90 | 63 | 13 |
| 119 | ICMP 100438 | 96 | 58 | 14 |
| 120 | ICMP 100439 | 82 | 61 | 12 |
| 121 | ICMP 100440 | 72 | 53 | 12 |
| 122 | ICMP 100430 | 87 | 41 | 11 |
| 123 | ICMR 100931 | 92 | 64 | 12 |
| 124 | ICMP 100431 | 80 | 48 | 11 |
| 125 | ICMP 100432 | 91 | 47 | 12 |
| 126 | ICMP 100433 | 109 | 59 | 12 |
| 127 | ICMP 100415 | 94 | 60 | 12 |
| 128 | ICMP 100416 | 89 | 58 | 13 |
| 129 | ICMP 100417 | 89 | 55 | 12 |
| 130 | ICMP 100418 | 86 | 48 | 11 |
| 131 | ICMP 100419 | 90 | 47 | 13 |
| 132 | ICMP 100420 | 88 | 52 | 13 |
| 133 | ICMP 100421 | 112 | 51 | 13 |
| 134 | ICMP 100422 | 77 | 46 | 10 |
| 135 | ICMP 100423 | 81 | 51 | 12 |
| 136 | ICMP 100424 | 101 | 61 | 12 |
| 137 | ICMP 100425 | 87 | 53 | 11 |
| 138 | ICMP 100426 | 84 | 62 | 11 |
| 139 | ICMR 100208 | 67 | 50 | 9 |
| 140 | ICMR 100236 | 114 | 73 | 14 |
| 141 | ICMP 100427 | 85 | 57 | 11 |
| 142 | ICMP 100429 | 116 | 71 | 13 |
| 143 | ICMB 100077 | 92 | 42 | 12 |
| 144 | ICMB 100633 | 53 | 37 | 10 |
| 145 | ICMB 100635 | 56 | 41 | 10 |
| 146 | ICMB 100636 | 63 | 56 | 10 |
| 147 | ICMB 100637 | 73 | 45 | 11 |
| 148 | ICMB 100638 | 79 | 51 | 12 |
| 149 | ICMB 100198 | 67 | 44 | 10 |
| 150 | ICMB 100639 | 37 | 28 | 11 |
| 151 | ICMB 100641 | 84 | 46 | 10 |
| 152 | ICMB 100642 | 69 | 33 | 10 |
| 153 | ICMB 100643 | 90 | 44 | 10 |
| 154 | ICMB 100644 | 99 | 49 | 11 |
| 155 | ICMB 100645 | 104 | 55 | 11 |
| 156 | ICMB 100617 | 110 | 54 | 12 |
| 157 | ICMB 100618 | 81 | 43 | 10 |
| 158 | ICMB 100619 | 60 | 42 | 9 |
| 159 | ICMB 100620 | 73 | 42 | 11 |
| 160 | ICMB 100621 | 83 | 47 | 12 |
| 161 | ICMB 100622 | 75 | 38 | 10 |
| 162 | ICMB 100623 | 69 | 40 | 11 |
| 163 | ICMB 100624 | 67 | 46 | 10 |
| 164 | ICMB 100625 | 82 | 44 | 11 |
| 165 | ICMB 100626 | 65 | 50 | 14 |
| 166 | ICMB 100627 | 75 | 47 | 11 |
| 167 | ICMB 100628 | 77 | 49 | 13 |
| 168 | ICMB 100629 | 81 | 52 | 10 |
| 169 | ICMB 100630 | 80 | 53 | 10 |
| 170 | ICMB 100631 | 102 | 49 | 10 |
| 171 | ICMR 100999 | 87 | 42 | 11 |
| 172 | ICMR 101000 | 90 | 51 | 10 |
| 173 | ICMR 101001 | 88 | 49 | 12 |
| 174 | ICMR 100544 | 88 | 58 | 10 |
| 175 | ICMR 100568 | 99 | 58 | 11 |
| 176 | ICMR 101003 | 90 | 43 | 12 |
| 177 | ICMR 101004 | 99 | 51 | 11 |
| 178 | ICMR 101005 | 89 | 43 | 12 |
| 179 | ICMR 101006 | 95 | 54 | 12 |
| 180 | ICMR 100550 | 81 | 49 | 11 |
| 181 | ICMR 101007 | 69 | 42 | 12 |
| 182 | ICMR 101008 | 102 | 47 | 13 |
| 183 | ICMR 100640 | 91 | 42 | 9 |
| 184 | ICMR 101010 | 102 | 57 | 12 |
| 185 | ICMR 101011 | 74 | 46 | 12 |
| 186 | ICMR 101012 | 97 | 51 | 13 |
| 187 | ICMR 101013 | 95 | 47 | 12 |
| 188 | ICMR 100556 | 73 | 41 | 10 |
| 189 | ICMR 101014 | 80 | 48 | 9 |
| 190 | ICMR 101015 | 85 | 44 | 12 |
| 191 | ICMR 101016 | 76 | 41 | 11 |
| 192 | ICMR 101017 | 89 | 50 | 13 |
| 193 | ICMB 100652 | 62 | 34 | 11 |
| 194 | ICMB 100653 | 60 | 43 | 10 |
| 195 | ICMB 100671 | 71 | 49 | 14 |
| 196 | ICMB 100463 | 55 | 41 | 12 |
| 197 | ICMB 100244 | 62 | 36 | 11 |
| 198 | ICMB 100302 | 43 | 21 | 10 |
| 199 | ICMR 100041 | 52 | 33 | 9 |
| 200 | ICMR 100990 | 49 | 30 | 9 |
| 201 | ICMR 1203 | 88 | 56 | 12 |
| 202 | ICMR 1501 | 91 | 40 | 12 |
| 203 | ICMR 1502 | 117 | 64 | 12 |
| 204 | ICMR 1503 | 94 | 48 | 13 |
| 205 | ICMB 1504 | 83 | 47 | 9 |
| 206 | ICMR 1505 | 72 | 37 | 10 |
| 207 | ICMR 1202 | 76 | 32 | 10 |
| 208 | ICMB 100195 | 82 | 42 | 11 |
| 209 | IPC 21 | 41 | 34 | 13 |
| 210 | IPC 492 | 48 | 28 | 12 |
| 211 | IPC 337 | 47 | 41 | 11 |
| 212 | IPC 616 | 85 | 62 | 13 |
| 213 | IPC 689 | 74 | 53 | 11 |
| 214 | IPC 716 | 57 | 30 | 10 |
| 215 | IPC 1027 | 41 | 29 | 10 |
| 216 | IPC 736 | 44 | 40 | 11 |
| 217 | IPC 774 | 82 | 66 | 13 |
| 218 | IPC 417 | 36 | 29 | 10 |
| 219 | IPC 835 | 39 | 33 | 12 |
| 220 | IPC 1047 | 35 | 28 | 10 |
| 221 | IPC 954 | 36 | 29 | 8 |
| 222 | IPC 957 | 43 | 27 | 11 |
| 223 | IPC 367 | 45 | 36 | 11 |
| 224 | IPC 1025 | 46 | 31 | 8 |
| 225 | IPC 1040 | 45 | 34 | 9 |
| 226 | IPC 1189 | 64 | 35 | 11 |
| 227 | IPC 1329 | 54 | 52 | 13 |
| 228 | IPC 1444 | 75 | 62 | 13 |
| 229 | IPC 1470 | 38 | 29 | 10 |
| 230 | IPC 1485 | 40 | 34 | 10 |
| 231 | IPC 1466 | 73 | 46 | 11 |
| 232 | IPC 1503 | 38 | 41 | 13 |
| 233 | IPC 1536 | 79 | 62 | 12 |
| 234 | IPC 795 | 46 | 29 | 11 |
| 235 | ICMB 92111 | 34 | 29 | 11 |
| 236 | ICMB 92888 | 44 | 29 | 15 |
| 237 | ICMB 96666 | 53 | 43 | 12 |
| 238 | ICMB 97111 | 51 | 28 | 12 |
| 239 | ICMB 98666 | 46 | 37 | 11 |
| 240 | ICMB 01444 | 61 | 40 | 13 |
| 241 | ICMB 01888 | 37 | 29 | 11 |
| 242 | ICMB 02555 | 52 | 43 | 11 |
| 243 | ICMB 03555 | 55 | 35 | 11 |
| 244 | ICMB 03666 | 51 | 45 | 14 |
| 245 | ICMB 04555 | 50 | 39 | 12 |
| 246 | ICMB 05666 | 57 | 40 | 12 |
| 247 | ICMB 06555 | 68 | 42 | 11 |
| 248 | ICMB 06888 | 47 | 39 | 12 |
| 249 | ICMB 07333 | 57 | 47 | 11 |
| 250 | ICMB 07777 | 53 | 33 | 11 |
| 251 | ICMB 07999 | 40 | 31 | 11 |
| 252 | ICMB 08555 | 55 | 24 | 10 |
| 253 | ICMB 10888 | 51 | 37 | 10 |
| 254 | ICMB 11555 | 50 | 40 | 11 |
| 255 | ICMB 11777 | 50 | 40 | 10 |
| 256 | ICMB 11999 | 37 | 27 | 12 |
| 257 | ICMB 14111 | 66 | 51 | 11 |
| 258 | ICMB 14444 | 52 | 49 | 12 |
| 259 | ICMB 15777 | 64 | 48 | 12 |
| 260 | ICMR 06999 | 37 | 33 | 11 |
| 261 | ICMR 07666 | 46 | 36 | 12 |
| 262 | ICMR 07777 | 50 | 48 | 11 |
| 263 | ICMR 07888 | 41 | 29 | 9 |
| 264 | ICMR 08111 | 32 | 27 | 8 |
| 265 | ICMR 08333 | 45 | 41 | 10 |
| 266 | IPC 186 | 64 | 48 | 12 |
| 267 | ICMR 08888 | 50 | 38 | 11 |
| 268 | ICMR 08999 | 43 | 34 | 11 |
| 269 | ICMR 09333 | 38 | 34 | 11 |
| 270 | ICMB 07888 | 70 | 36 | 11 |
| 271 | ICMR 09666 | 45 | 37 | 13 |
| 272 | ICMR 10222 | 37 | 19 | 9 |
| 273 | ICMR 10888 | 42 | 35 | 8 |
| 274 | ICMR 11666 | 34 | 25 | 9 |
| 275 | ICMR 12333 | 54 | 33 | 16 |
| 276 | ICMR 12444 | 46 | 37 | 13 |
| 277 | ICMR 12777 | 36 | 41 | 9 |
| 278 | ICMR 16111 | 32 | 25 | 9 |
| 279 | ICMR 16555 | 82 | 45 | 10 |
| 280 | ICMR 16888 | 36 | 39 | 12 |
| 281 | ICMR 16999 | 39 | 30 | 11 |
